# Shifting the paradigm of research-to-policy impact: Infrastructure for improving researcher engagement and collective action

**DOI:** 10.1017/S0954579424000270

**Published:** 2024-03-22

**Authors:** Taylor Scott, Max Crowley, Elizabeth Long, Brandon Balma, Jessica Pugel, Brittany Gay, Angelique Day, Jennie Noll

**Affiliations:** 1 Edna Bennett Pierce Prevention Research Center, Pennsylvania State University, State College, PA, USA; 2 Department of Political Science, University of Minnesota, Minneapolis, MN, USA; 3 School of Social Work, University of Washington, Seattle, WA, USA,; 4 Department of Psychology, University of Rochester, Rochester, NY, USA; 5 Mt. Hope Family Center, Rochester, NY, USA

**Keywords:** evidence-based policy, research impact, research translation, science policy

## Abstract

The body of scientific knowledge accumulated by the scholarly disciplines such as Developmental Psychopathology can achieve meaningful public impact if wielded and used in policy decision-making. Scientific study of how policymakers use research evidence underscores the need for researchers’ policy engagement; however, barriers in the academy create conditions in which there is a need for infrastructure that increases the feasibility of researchers’ partnership with policymakers. This need led to the development of the Research-to-Policy Collaboration model, a systematic approach for developing “boundary spanning” infrastructure, which has been experimentally tested and shown to improve policymakers’ use of research evidence and bolster researchers’ policy skills and engagement. This paper presents original research regarding the optimization of the RPC model, which sought to better serve and engage scholars across the globe. Trial findings shed light on ways to improve conditions that make good use of researchers’ time for policy engagement via a virtual platform and enhanced e-communications. Future directions, implications, and practical guidelines for how scientists can engage in the political process and improve the impact of a collective discipline are also discussed.

## Introduction

In 2004, Dante Cicchetti wrote in his then 30-year reflection, “An Odyssey of Discovery”, about the state of our field and the realities of getting scientific solutions used by policymakers:

“*As the new millennium dawned, there was cause for much optimism with respect to the understanding of child maltreatment. We possessed more knowledge regarding the etiology and sequelae of this social ill than at any other point in history. Researchers, practitioners, and policymakers were well positioned to implement prevention and intervention programs that could stem the increasing incidence of child maltreatment, ameliorate its harmful biological and psychological sequelae, and promote resilient adaptation. However, economic and political forces were operative that highlighted how long we have to go before our journey concludes successfully. Decreased financial resources attributable to economic uncertainties, war expenditures, homeland security costs, and political ideologies began to erode resource allocation for both research and service provision. Despite accruing evidence on effective prevention and intervention strategies, the United States appears to be falling farther behind in its efforts to combat child abuse and neglect. In view of the failure of society to effectively stop the occurrence of child maltreatment, current policies and practices must be examined*.” (Cicchetti, 2004)

Now, 20 more years have passed and much of these frustrations still ring true to many researchers in our discipline. The need for effective solutions for improving the use of research in public policy remains a pressing issue. However, the grim reality of how science actually gets used in policy circles could easily cast gloom over the passions of the well-meaning scientist. In reality, profitable industries are the large drivers of policy decisions whereas cost-saving, evidence-based practices receive low uptake in policymaking and thus rarely reach the masses. Although the voices of diverse actors, including experts of a scientific discipline, are critical to sound policymaking, the absence of agenda-neutral research messengers hinders uptake of evidence-informed practices. It became clear during the COVID-19 pandemic that scientific fact alone is insufficient for guiding effective policy decision-making. Neutral, fact-based information is scarce in the policy process, which heightens the political fever pitch and erodes democracy through unchecked misinformation.

Even as academic Deans and Directors increasingly call for “impact” to be considered in faculty tenure and promotion guidelines with weight equal to research, teaching, and service, support for achieving measurable impact continues to be nebulous (McBride et al., 2019). So how do we harness this well-meaning enthusiasm while upholding realistic expectations of achieving a science impact? How do we embolden the next generation to continue to cling to the scientific method as viable means of improving public discourse? How do we encourage the types of impact activities that university leadership and funders value?

One strategy is to encourage scientists to cultivate their program of research by addressing policy-relevant questions. Having a finger on the pulse of what constitutes ‘relevance’ will necessarily require moving beyond one’s isolated laboratory and toward a collective understanding of how policymakers might address societal problems. In this paper, we explain how academic researchers can have the greatest public impact if they are part of a collective discipline rather than operating in isolation. Instead of merely uplifting single studies or one’s own authored papers, the body of knowledge amassed by a scientific discipline is much more apt for policy impact. For instance, Developmental Psychopathology, as championed by Dante Cicchetti and colleagues (e.g., Cicchetti & Toth, 2009), is recognized as a scientific discipline focused on holistic theory of integrated, dynamic systems along a continuum between the normal and the abnormal, testing mechanistic hypotheses about adaptive processes and novel interventions. Through this lens of individual variability and multiple levels of analysis, a host of developmental conditions (e.g. child maltreatment, substance misuse, violence) began to be studied from a strength-based perspective as opposed to one of pathology, thus providing a platform for combating stigma, understanding both multi- and equifinality, articulating the interplay between biology and behavior, and promoting models of resilience. Identifying with, being influenced by, and reading literature disseminated from a collective discipline such as Developmental Psychopathology will not only help researchers be versed in potential policy-relevant topics on which to base a program of research, but will challenge them to think collectively about how the research of their discipline can impact policy.

Being steeped in an identified discipline enables one to become an effective steward of the scientific disciplinary knowledge. This is greatly facilitated by the effective culmination and curation of relevant research and published articles. The flagship journal of Developmental Psychopathology, *Development and Psychopathology* is one such place. Executive Editor since its inception in 1989, Dante Cicchetti has been instrumental in forging the discipline of Developmental Psychopathology. The journal constitutes the culmination of original research articles, literature reviews, meta-analyses, and special issues that exemplify, elevate, and constitute a blueprint for the entire discipline. With generous page-limits, the journal offered space to expound on relevance to policy and practice, enhancing the usability of content for research translation. Moreover, *Development and Psychopathology* is known for its scientific rigor. As such, articles published in *Development and Psychopathology* are of the highest quality and are trusted sources for innovation, unassailable methods, strong causal inference, and translatability – ever-important qualities in research used to inform policy.

In tribute to the half century of work in the field – and continued struggles to see our science used by policy communities – we describe a strategy for systematically supporting the use of science in public policy by organizing scientific disciplines known as the Research-to-Policy Collaboration (RPC) model (Crowley et al., 2018, 2021). The RPC model provides an enabling environment for researchers to engage and coalesce in their discipline through a ‘Rapid Response Network’ (RRN), teaches researchers how to translate their work for policy-relevance, facilitates policy engagement, and encourages researchers to develop policymaker partnerships (TrestleLink, n.d.). We recognize, however, a policy engagement infrastructure is necessary for researchers to engage in these processes. Unfortunately, most researchers lack the time to devote to policy engagement, citing the enduring and competing demands of teaching, research, and service that invariably take priority in the quest to achieve tenure and promotion. Yet, there are increasing expectations in most academic departments that faculty demonstrate the impact of their work at the level of practice and/or policy relevance as well as by having a voice or a presence on the policy stage.

In light of the contextual backdrop that hinders researchers’ policy engagement, we present new data from a study that sought to optimize the RPC to better serve and engage scholars. Specifically, the study sought to understand ways in which boundary spanners (i.e., RPC implementers) could leverage a virtual platform and facilitate collective action among scholars. Finally, we discuss future directions and implications for both the RPC model optimization and individual scholars who seek to improve the public benefit and impact in their work.

### Theoretical foundations of the Research-to-Policy Collaboration

Scholars studying effective science translation bemoan the lag between scientific discovery and its implementation in policy or practice (Morris et al., 2011). Here we argue that the “gap” reflects a systemic issue in which there is inadequate translation infrastructure that supports the usability of scientific knowledge. First, we will explain the science behind how research is used by policymakers, and then explain the systemic barriers within the academy that hinder our progress.

Fundamental research from the 1970’s provides us with core theory that guides the field studying policymakers’ use of research evidence (URE) today. The gap between research and policy is driven in large part by a lack of interpersonal ties between professional communities (Boaz et al., 2019; Bogenschneider et al., 2019; Bogenschneider & Corbett, 2021). There is typically infrequent contact between actors comprised in “two communities” of research and policy, which operate in “parallel universes” because they are characterized by different values, norms, routines, and languages that hinder our connectivity (Bogenschneider & Corbett, 2011; Brownson et al., 2006; Caplan, 1979). This is problematic because although research can “enlighten” the way policymakers think about issues, evidence use can be highly interactive, especially since policy problem solving is complex and influenced by myriad political considerations and since research interpretation is a formative and iterative process (Kingdon, 1984; Mackie et al., 2015; Weiss, 1979).

Substantial research since then has repeatedly demonstrated that relationships and collaborations between our communities can facilitate policymakers’ URE (Boaz et al., 2019; Oliver et al., 2014). Collaboration is potentially a pathway for making evidence more usable and relevant in real time policy initiative (Bogenschneider, 2020; Mackie et al., 2015; Scott et al., 2022). Better understanding the needs of the hoped for users of research also increases the likely impact of research questions we are asking. This body of scholarship additionally highlights the need for scientists to work with decision makers as “honest brokers” who maintain scientific integrity and political neutrality by impartially discussing a variety of policy options (Pielke, 2007). The honest brokerage concept also differentiates outreach strategies by envisioning a politically neutral, educational, and advisory role rather than a role of lobbying (i.e., opposing or supporting specific legislation) or political activism (e.g., applying pressure to advance a political agenda). Policy skills or “know how” (Day et al., 2019) also play a role in the effectiveness of researchers’ policy efforts, especially skills that mitigate “clashes” between communities by reducing miscommunication and mistrust (Bogenschneider et al., 2013; Brownson et al., 2006; Scott et al., 2019).

A primary conclusion from this body of work is that researchers need to develop trustworthy partnerships by engaging in the policymaking process if we expect evidence to be used. Although researchers’ policy engagement is a critical component for bridging research and policy, they face numerous barriers. As previously stated, “the most substantial impediments to researchers’ policy engagement are systemic and institutional in nature” (Scott et al., 2019). The systemic nature of this challenge is illustrated in scholarship investigating barriers to researchers’ public engagement (Guillot-Wright & Oliver, 2023; Kim et al., 2023), leading scholars to call for systemic changes and improved infrastructure (Boaz et al., 2019; Gamoran, 2019) as well as active efforts to “modernize scholarship” (Aurbach et al., 2023). These barriers include high institutional demands on their time, limited reward recognizing policy work, minimal resources or compensation for policy tasks, and few opportunities to access policy spaces or train on how to navigate those spaces (Kim et al., 2023). Limited reward, time restraints, limited training or navigational know-how are repeatedly mentioned in the literature as barriers to researchers’ policy engagement (Bogenschneider et al., 2013; Day et al., 2019; Fisher-Borne et al., 2015; Gollust et al., 2017; Guillot-Wright & Oliver, 2023; Jones et al., 2009; Sisk et al., 2011). Moreover, there is a need for “boundary spanners” who connect producers and users of research since these tasks are not typically codified into academic job responsibilities or promotion and tenure (Bell & Lewis, 2023; Goodrich et al., 2020). Despite these barriers pointing to systemic challenges that researchers face within academia, institutions struggle to conceptualize and design infrastructure to support appropriate scholarly policy engagement (Gamoran, 2018; Hopkins et al., 2021).

Some argue that infrastructure that facilitates “brokerage” between communities is needed to systematically traverse these communities to cultivate connections between scientists and policymakers so that evidence can be more useful at critical times, which are seen as facilitators to the use of evidence in policy (Boaz et al., 2019; Cairney & Oliver, 2020; Gamoran, 2019; Oliver et al., 2014). Boundary spanning entities may be well positioned to connect researchers and government officials (Gamoran, 2018) and are gaining recognition for their ability to facilitate trusting relationships between scholars and government officials (Franklin et al., 2019). However, limited systematic investment in brokerage infrastructure perpetuates the disconnect between research producers (e.g., academics) and end users (e.g., policymakers), which ultimately reduces the public benefit of science (Gamoran, 2018; Goodrich et al., 2020). One challenge has been to develop infrastructure within a fragmented research ecosystem that offers little support or recognition for translation and routinely reinforces self-aggrandizing independence. Boundary spanning infrastructure must support the collective action of researchers in a discipline and operate within the time constraints of scholarly pursuits.

### Boundary spanning infrastructure

**Figure 1.**
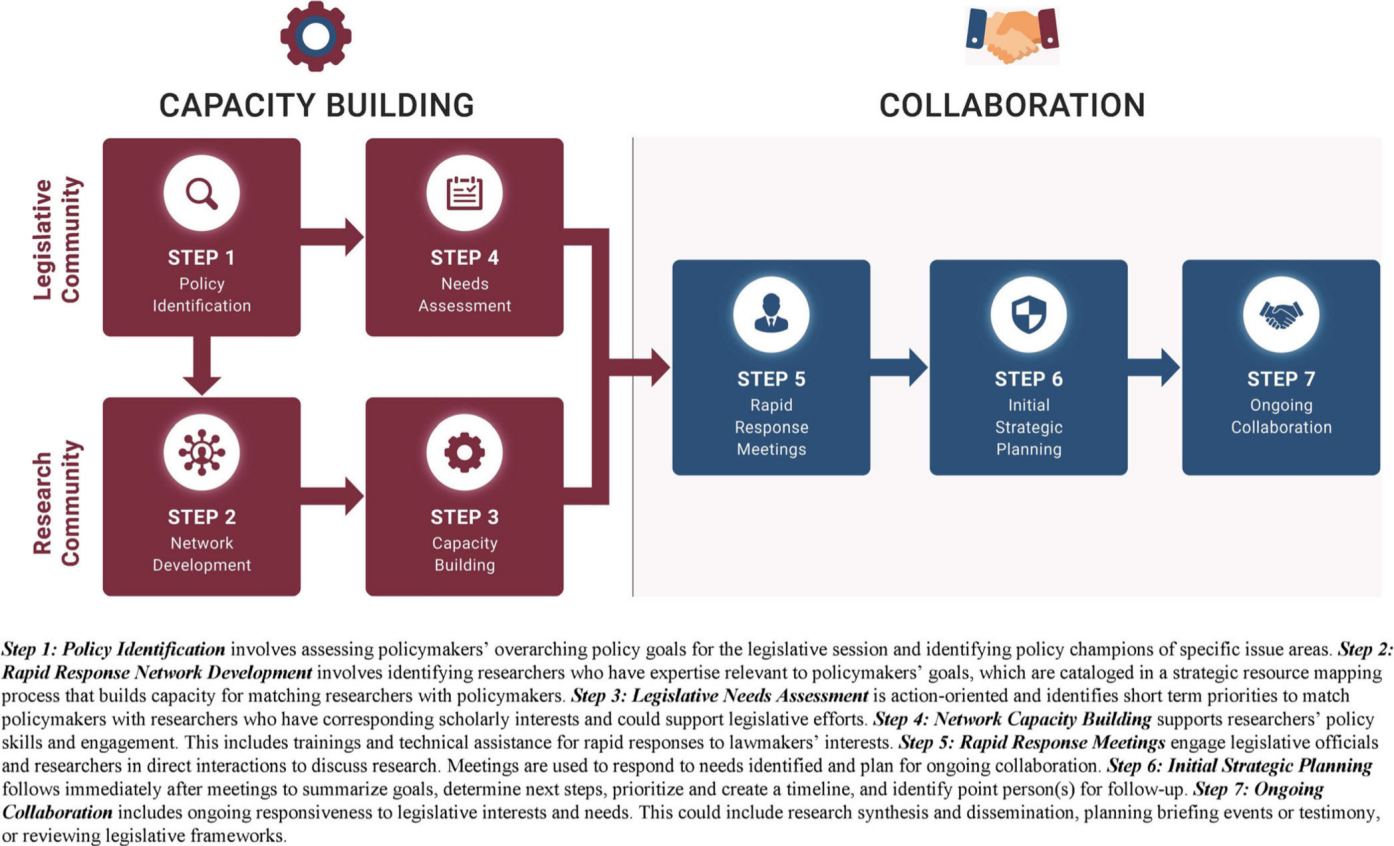
Model Steps for the Research-to-Policy Collaboration.

The Research-to-Policy Collaboration (RPC) model was developed in light of the need for infrastructure that mitigates structural barriers hindering scholars’ policy engagement. Thus, the model aims to make it feasible and efficient for researchers to achieve policy impact by providing administrative support to schedule meetings with policymakers who share related interests. Model steps are illustrated in Figure 1. Skilled boundary spanners (i.e., policy associates) facilitate meetings between the “right policy-maker” and the “right researcher” at the “right time”, which mitigates researchers’ concerns by supporting the effective use of time (Jones et al., 2010). This model cultivates nonpartisan policy engagement by listening to legislators’ needs for evidence before facilitating a response among researchers within a discipline. Researchers are trained to engage as honest brokers without lobbying (Crowley et al., 2018). The RPC democratizes scholarly policy engagement by mobilizing the scientific community to support policymakers’ evidence needs. An open platform is intended to counteract gatekeeping of what research and which scientists are represented in policy decisions. Focusing on the end-user’s strategic evidence needs guides a collective response from scholars within a discipline studying issues on the current policy agenda.

**Figure 2.**
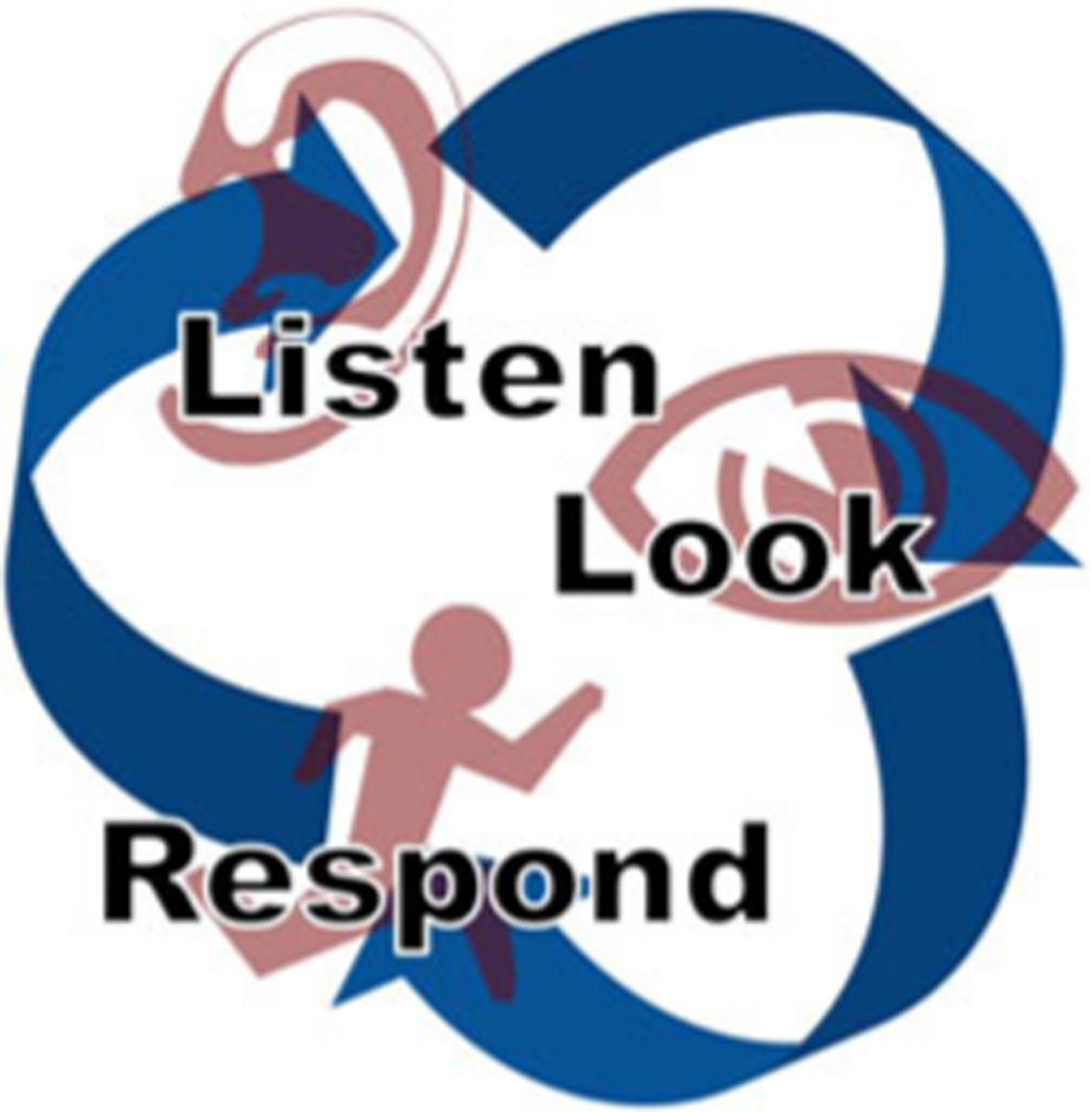
Rapid Response Process of the Research-to-Policy Collaboration.

A feedback loop (Figure 2) illustrates the cyclical nature of engaging the RRN for developing researcher-policymaker collaborations. Central to the model is to first *listen* to policymakers’ goals, priorities, and understand their need for research evidence (e.g., etiology, examples of successful interventions or challenges in practice) through a legislative needs assessment. Then, we *look* for researchers to *respond rapidly* to those interests. This often results in requests for production of policy briefs and factsheets, congressional briefings and testimony, and requests to review or provide legislative language for bill drafting (Crowley et al., 2018). The response cycle repeats upon the reception of policymaker feedback about how researchers can continue to support their policy goals, and scholars in the discipline are continuously engaged in ongoing relationship development and strategic support of the office’s evidence needs. A series of seven steps illustrates the replicable process for capacity building and collaboration (Crowley et al., 2018, 2021).

Core to RPC implementation is the development of the RRN. It is critical to develop a remote-based infrastructure for organizing a scientific discipline and provide a feasible and efficient pathway for researchers across the United States and worldwide to engage in policy efforts. The online RRN infrastructure also involves cataloging participants’ areas of expertise so they may be contacted via email when their interests match those of participating policymakers. Thus, researchers involved in the RRN receive tailored opportunities to engage in the policy process when there are opportunities related to their areas of expertise. Researchers are provided with training and technical assistance that supports appropriate information exchange (i.e., no lobbying). Participants routinely respond to policymakers’ interest areas via email and during meetings (online or in-person) in which policymakers are matched with researchers who share interests. The legwork of cultivating policy opportunities, organizing research communications, and scheduling meetings is managed by trained boundary spanners (i.e., policy associates), which makes it more feasible for researchers to participate with limited time availability. Moreover, since the responses are curated from a network of scholars within a discipline, the burden of the research translation is shared among colleagues within a field. Since the diversity of voices represented in the RRN affect the message received by policymakers, it is critical that the RRN is populated by scholars who range in expertise in the discipline. This component of the model is critical for rapidly responding to policymakers’ goals and interests, and thus its ability to be timely, responsive, and precise is contingent on the makeup of a scholarly discipline that is at the ready for research translation and impact.

### Empirical evaluation of the RPC

#### Prior evaluations of the RPC model

Both a pilot and randomized controlled trial of the RPC model indicate feasibility and impact of bringing researchers and legislative officials together to translate research for policymaking. Never before have solutions for increasing evidence-informed policymaking been experimentally proven effective. A robust data backbone behind the RPC model implementation has enabled the study of research translation and policy impact. This includes studies related to feasibility and cost of the model’s implementation (Crowley et al., 2018), benefits for researchers’ skill development (Scott et al., 2019) and policy engagement (Crowley et al., 2021), and impact on congressional policymakers’ URE in legislation (Crowley et al., 2021). The RPC model was first evaluated within a feasibility trial of a pilot implemented by the National Prevention Science Coalition and supported by the Doris Duke Charitable Trusts, then later brought to scale in the US Congress during an experimental evaluation supported by NICHD and the WT Grant Foundation. Results of the feasibility study showed that policymakers demonstrated an appetite for research and interactions with researchers in the 114^th^ Congress, which resulted in matching researchers with ten congressional offices and producing a total of 79 unique requests for child- and youth-oriented evidence (e.g., briefs, speakers, policy analysis; Crowley et al., 2018). The legislative staff’s time commitment demonstrated their demand for obtaining research insights from honest brokers and their willingness to collaborate when it aids staffers’ current demands. Thus, this is why it is so critical that the RPC connects legislative offices with researchers based on current policy priorities and legislative activity. The congressional randomized controlled trial (RCT) of the RPC model occurred in the 116^th^ Congress and results indicated that RPC supported offices were over 20% more likely to write and introduce child and family bills that included research evidence than control offices. Specifically, we found that legislators were not introducing *more* bills, they were just more apt to use evidence language in the bills they were introducing. This finding is consistent with the notion of supporting legislators’ existing goals instead of lobbying for specific bills. Further, offices randomized to receive the RPC reported over 7% increase in their value for scientific evidence. Importantly, we found no evidence that the RPC increased tactical (political) URE, which is historically prone to misuse (Doucet, 2019; Kirkland, 2019). An independent ethnography found offices receiving the RPC valued the opportunity to work with researchers directly, found RPC research responses helpful, and planned to continue engaging with researchers in the future (Guillot-Wright & Oliver, 2020). In subsequent experimental trials testing the effect of RPC’s research dissemination among state legislators, results indicated that those randomly assigned to receive RPC supports referenced research in over 20% more of their social media posts compared to control legislators (Scott et al., 2023). In sum, studies provide evidence of the feasibility and value of engaging policymakers in the model alongside evidence of effectiveness.

Prior work has also examined the experiences of researchers engaging in the RPC model. The feasibility study highlighted researchers’ substantial time commitment to voluntarily share their research expertise, which demonstrated the untapped desire for engaging in policy opportunities among participants. It was estimated that around 30 researchers in the RRN spent roughly 288 hours engaging in rapid responses to legislative requests for evidence. Time commitment was roughly 6 hours for participants of the RRN unless they also participated in an event to meet staffers, in which case ten researchers spent roughly 70 hours of direct interaction with offices (Crowley et al., 2018). Pilot survey data also highlighted measurable benefits regarding improvements in researchers’ reported policy skills and self-efficacy (Scott et al., 2019). Subsequently, the congressional RCT also studied the experiences of researchers and found that researchers randomized to receive the RPC had a greater level of policy engagement than the control group, increased policy knowledge, and reported that policy engagement improved their own program of research. Many of these effects were driven by the experiences of scholars of color (Crowley et al., 2021).

Finally, the prior cost analyses of RPC implementation are relevant to the current study aims of optimizing the model in ways that improve the value of researchers’ time and streamline the communications and effort of policy associates. The pilot data indicated the feasibility to sustainably operate this model within academic funding models. Initial cost-effectiveness analyses indicate that the RPC is an efficient approach to legislative outreach compared to traditional advocacy and lobbying costs. The costs are feasible for support by professional societies, nonprofit research institutes, and university units (Crowley et al., 2018). These analyses revealed it costs about $1,600 to provide the RPC to a congressional office and it costs approximately $444 to elicit a request for scientific evidence to support policy development. In sum, while the RPC model is feasible to implement within a modest price point, investigators continued to seek ways to streamline and improve the researcher experience that is so critical for implementation success and sustainability.

### Areas of improvement

While the prior studies shed light on the cost and feasibility of implementation alongside measurable impact on the research community and policy, there was a need for implementation research that could shed light on ways to optimize the model. There were several pain points felt in the implementation of the RPC model by both the policy associate team and the participating scholars. Top of mind was how to effectively communicate in a digital and geographically dispersed environment in which email traffic was the lifeblood of researchers’ policy engagement experiences. Not only was there a need to improve communication with and between RRN participants, but the challenge was exacerbated by a need for succinct and targeted messages, as well as reducing the frequency in demands for participants’ attention. For instance, we recognized that participating researchers desired more transparency, better communication, improved networking, recognition, and other features (Gay, 2018); however, the risk of increasing back-end operations involved cannibalizing effort and time for meaningful responses to policymakers’ questions.

Another pain point relates to recruiting researchers to the RRN since if the network isn’t populated by a diverse array of scholars, then the resultant policy responses are less relevant and precise, thus yielding responses that are less valuable to participating policymakers. Reducing time demands on participants could increase the accessibility of the model for engaging a wider and more diverse array of actors within a scholarly discipline. Communicating this feasibility during RRN recruitment was yet another challenge. Moreover, timeliness is critical for improving impact, and any efforts to streamline the implementation burden might also reduce the cost of policy associate time, improving the cost-effectiveness of the model. Thus, the gaps in knowledge to date involve improving processes of researchers’ collective engagement in the virtual RRN to improve the value and efficiency of their time, implementers’ time, and overall agility and impact.

### The current study: optimizing researchers’ policy engagement experiences

Findings from evaluating the RPC model suggest that it is possible to accelerate the influence of Developmental Psychopathology by improving policymakers’ URE. Despite its measurable successes, investigators sought to continue refining and improving the model’s ability to support the efficiency and value of researchers’ policy engagement experiences. In particular, there was a need for making the model more agile and timely to improve the efficient use of scholars’ time commitment as well as reduce the cost of implementation, making it more streamlined and sustainable. In this paper, we present original study data derived from rapid-cycle randomized controlled trials that aimed to shed light on ways to support researchers’ policy engagement. Embracing implementation science for understanding model processes and participant experiences, we aimed to test actionable strategies that enriched researchers’ engagement in the RPC model. Not only does scholarly policy engagement provide a useful indicator of the model’s benefit to the research community, it is also a proximal indicator of timely responsiveness to policymakers’ interests, which is fundamental to the effectiveness of the RPC model.

We defined a successful optimization as the extent to which researchers engaged more often within a proper timeframe or in more meaningful ways, or reported other benefits from their engagement. Timeliness of response is critical for policymakers’ URE (Boaz et al., 2019; Bogenschneider & Bogenschneider, 2020; Brownson et al., 2006; Choi et al., 2005; Kingdon, 1984; Mackie et al., 2015; Oliver et al., 2014). Researchers’ rate of engagement also suggests improvements in their participation experience. Consistent with empirical literature (Guillot-Wright & Oliver, 2023; Kim et al., 2023; Sisk et al., 2011), RPC researchers have previously noted a lack of time as the most salient barrier to their participation (Gay, 2018). The perceived benefits of engagement must outweigh the costs (e.g., time, loss of control; (Kanter & Fine, 2010; Koh et al., 2007). Therefore, structures that improve the time-efficiency of policy engagement may enhance the timeliness and impact of bridging activities. In this study, we consider a continuum of engagement behaviors consistent with a “ladder of engagement” (Kanter & Fine, 2010), in which enrollment into the network constitutes a foot-in-the door, entry-level engagement behavior, whereas deeper engagement occurs over time by participating in rapid responses that address policymakers’ interests.

Given the salience of researchers’ engagement for the model’s optimization, a review of plausible mechanisms for enriching scholarly policy engagement was conducted. These hypothesized predictors correspond with ecological levels of analysis, including individuals’ motivations, attributes of the organization or its leadership, or characteristics of the policy network as a whole. Our theoretical framework draws upon principals of empowerment and community organizing – where strategies are employed to fundamentally change relevant structures that ensure equitable distribution of resources and power by developing network relationships to mobilize collective action (Hale, 2014). Empowering settings strengthen the capacity for participation of group members, facilitate access to resources, and create structures for shared decision-making (Foster-Fishman et al., 1998; Israel et al., 1998). Similarly, coalescing researchers into coordinated networks or coalitions has the potential to generate collective action through a shared vision, joint strategy, and collaborative leadership (Himmelman, 2001; Wolff et al., 2016). Specifically, a sense of community may help to develop a critical mass of individuals who collectively sustain work toward goals they perceive as aligned with others in the network or community (Brady et al., 2015; Bussell & Forbes, 2002; Carlsson, 2000; Heylighen et al., 2013; Sandström & Carlsson, 2008; Wasko & Faraj, 2005). We also recognize that the RPC implementation approach is highly interpersonal, not just between researchers and policymakers, but between researchers and with the implementation leadership as well. Thus, we sought to understand strategies for bolstering a collective response by cultivating a sense of community (e.g., shared purpose), tools and information for independent navigation, positive reinforcement via recognition, and development of relationships with implementation leadership and between network members.

Since the implementation of the RPC model involves organizing and mobilizing a geographically dispersed network of researchers, our optimizations primarily pertain to developing an empowering setting online and improved e-communications. The need for improving the experience of remote participants, alongside theoretical conditions for effective community organizing, led us to focus on two areas of optimization: a virtual policy network and leadership communication.

### Virtual policy network

Intuitive policy tools have the potential for supporting timely engagement by reinforcing the structure of the RPC’s RRN, as well as supporting individual and collective efficacy. We launched a digital platform to provide a setting to develop a virtual community and hub for policy engagement. We believed that an online space could address geographical dispersion, a barrier to engagement (Irvin & Stansbury, 2004). We also hoped that engaging researchers from their home offices could sustain high-impact work even during times of national crisis, as well as supplement in-person interactions. Since our goal was primarily to provide a virtual space for collective action and cultivate a sense of community, we built an interactive online platform that featured a forum and personalized user profiles. We also sought to foster engagement by embedding tools and trainings for navigating the policy arena. Although virtual advocacy platforms have shown great promise for amplifying grassroots engagement with policymakers in other domains (Johansson & Scaramuzzino, 2019; Jordan, 2010), little is known about how to mobilize researchers specifically – and none have been experimentally evaluated. Such an infrastructure may provide “deliberative social spaces that allow routine engagement” (*Vivian*’*s Voice*, 2019) among researchers with policymakers as well as increase independent and sustainable policy engagement. Previous work with non-researchers indicates that virtual platform engagement is dependent upon (1) positive user experience – including intuitiveness, simplicity, responsiveness, and interactivity (Heylighen et al., 2013; Koh et al., 2007; Ziaie & Krcmar, 2014); (2) persuasive technology tactics such as goal setting, visual feedback, behavioral triggers, reward systems (e.g., peer evaluation), and gamification (e.g., symbolic rewards, levels, and votes; Heylighen et al., 2013; Koh et al., 2007); and (3) facilitating network development comprised of social connections (Koh et al., 2007) and leadership roles within network sub-groups (Hale, 2014; Rappaport, 1987). We also sought to further explore opportunities to increase recognition and reward for policy engagement since academics are reinforced for professional reputation and status (Blackmore & Kandiko, 2011; Kanter & Fine, 2010; Wasko & Faraj, 2005; Ziaie & Krcmar, 2014), whereas a lack of award and recognition has been noted as salient barriers to policy engagement (Bell & Lewis, 2023; Goodrich et al., 2020; Kim et al., 2023; Scott et al., 2019).

*Leadership Communication* involves strategic messages that position a group toward a shared goal (Barrett, 2008; Stone, 2002; de Vries et al., 2010) by shaping the purpose, vision, and roles of group members (Koh et al., 2007); fostering trust (Wolff et al., 2016); and recognizing the abilities of group members (Rappaport, 1987). The RPC optimization goals involved improving e-communications to promote engagement and attachmentto the network by reinforcing a sense of community (e.g., shared identity and emotional connection, making benefits more explicit, indicating ways that the group has collective power) (McMillan & Chavis, 1986; Sarason, 1974). Deliberate phrasing of an intermediary’s messages may also change the way people think about each other and even themselves (Rappaport, 1987) or mobilize action (Lovejoy & Saxton, 2012). In thiscase, messagesmay elicit researcherpolicyengagement; however, little is known about what messages researchers will find motivating or will achieve collective action. We hypothesized that communicating in ways that resonated with researchers’ motivations and their experiences in the setting would be critical (Brodsky & Cattaneo, 2013; Maton, 2008). Early work suggested that individuals may be motivated by their perceived influence or effectiveness and opportunities for developing policy-related efficacy (Gay, 2018; Kanter& Fine, 2010; Weible, 2016). We also expected thatpersonable communication strategies that applied positive reinforcement (Heylighen et al., 2013) gratitude expressions, (Grant & Gino, 2010), and reinforced shared sense of purpose or value of research impact would be effective (Stone, 2002; Weible, 2016; Zhou, 2011). Lastly, we aimed to support transparency in the process to build trust (Heylighen et al., 2013) as was also recommended in prior feedback from participants (Gay, 2018). In sum, deliberate forethought and critical analysis is central to developing effective messaging and should be inspired by the motivations and incentives that strengthen knowledge translation capacities (Jones et al., 2010).

The current study aimed to investigate the effectiveness of theoretical optimizations that could enhance the timeliness and efficiency of researchers’ policy engagement. Our primary aim was to enhance researchers’ experiences related to their participation in online rapid responses to congressional requests pertaining to children and families.

## Methods

A mixed method evaluation involving field experiments and focus groups was used to study the effectiveness of optimizations on observable policy engagement behaviors of researchers. Consistent with design thinking methodology (Tantia, 2017), we sought to understand and improve the user experience of the RPC model’s virtual rapid response process and e-communications. In this case, the users and study sample comprised researchers participating in the RPC’s Rapid Response Network. Design thinking allowed us to identify and create solutions from the user-perspective, which was expected to aid in the application of innovative technologies in a virtual community (Brown & Wyatt, 2010). This involved better understanding researcher participants’ use of the virtual platform, challenging our assumptions regarding routine processes, redefining problems based on the user experience, and testing alternative solutions with field experiments. Thus, field experiments tested user behavior in different conditions, and feedback related to the results was elicited via focus groups, which then informed design improvements and repeated cycles of testing and optimization. Focus groups were particularly useful for understanding the user experience (i.e., researchers) in the virtual platform by asking about usability, challenges, features (including gamification badges and policy tools), and awards made to top contributing researcher participants. Ultimately, mixed methodologies allowed investigators to produce data that informed the development and refinement of optimizations for enhancing scholarly policy engagement. We conducted a total of 23 rapid-cycle experiments and four focus groups for this study and our methodologies are summarized below. The methodologies, including samples and outcome metrics, of each field experiment varied depending on the testing aim and the stage of the implementation process. For this reason, we provide detailed methods for each experiment in the appendix and summarize the different types of methods below.

Rapid-cycle experiments were used to understand the impact of implementation processes in a consolidated time period (Cody & Asher, 2014; Peek et al., 2014). The quantifiable data obtained in these processes involved observations of naturally occurring behavior, including digital engagement metrics. Data were passively collected, requiring no action from participants. Being generally considered low burden and innocuous, missing data were minimal. There were two methods of testing leadership communication, described here as *simultaneous messages* and *repeated messages* (Table S.1. summarizes these method descriptions), alongside a *structural* experimental method of testing the virtual policy network features.

In most of the leadership communication tests, *simultaneous messages* were used as long as it was appropriate to send a single, bulk message and there was a large enough sample with sufficient power to detect differences between groups randomly assigned for A/B message conditions. Emails were sent using a Client Relationship Management software that allowed the study team to track open and click rates using an invisible one-pixel image in line with the industry standard and unique URL hyperlinks for collecting individual-level engagement data. When relevant, actual follow-through such as network enrollment or event participation was tracked using internal records for completion of enrollment forms.

*Repeated messages* were conducted when examining researchers’ responses and contributions in the Rapid Response (RR) process. Their contributions were coded by project personnel. Each RR was sent to 10–*5*0 researchers; thus, to reach an adequate sample size, we repeated the message test across multiple RRs and used a mixed-effects design wherein recipients for a given RR solicitation were iteratively randomized to experimental message conditions. The field experiment was repeated until the sample size reached approximately 500 observations, a sample size determined in an a priori power analysis. This design allowed the study team to investigate the impact of messaging during the rapid response process since individual researcher participants typically received multiple solicitations.

A final methodology involved *structural experiments,* which tested the impact of tools or processes in the virtual platform as opposed to messages conveying leadership communication. This allowed the investigators to study the effect of the platform, gamification, policy tools, transparency procedures, and award procedures on observed engagement in the platform, RRs, and messages. Unlike the leadership communication tests, the observed effects on engagement patterns were expected to occur over time as opposed to immediate reactions to prompts for action.

Analysis of rapid-cycle data involved using logistic and negative binomial regression models that estimated the change in odds or frequency of opening an email, responding to emails, clicking embedded links, contributing to RRs, or using the platform. Nested designs and zero-inflated models were used when appropriate for the data structure (Erie et al., 2015; Ridout et al., 2001).

Focus groups were conducted with purposive samples of study participants based on their assignment to specific optimizations, prior to expanding those features to other RPC participants. Detailed sample characteristics and questions are provided in Appendix 1. The nature of the questions asked were informed by the results of the rapid-cycle field experiments. These included four focus groups that asked participants about their experiences in the platform, receiving badges (i.e., gamification), accessing policy tools pages, and receiving a Policy Scholars Award. Each focus group was guided by a set of open-ended questions and facilitators probed with clarifying questions. Focus groups were conducted in Zoom and the transcripts of recordings were used as study data for grounded theory analysis (Creswell & Poth, 2016; Strauss & Corbin, 1990). This involved inductive coding by two study team members who categorized concepts emerging from the data into themes (Strauss & Corbin, 1990). This process began with open and exploratory coding emerging from the data to develop theories about the participants’ experiences that could be further tested in rapid-cycle experimental trials.

## Results

### Virtual platform

We found that there was roughly equal engagement between researchers who were enrolled in the platform compared to those who responded to Rapid Responses via email (Appendix 2). However, focus group findings shed light on how the platform benefited researchers’ efficiency in contributing to rapid responses (Appendix 1). The platform allowed participants to be relatively aware of one another’s contributions, which reduced redundancy between replies. Focus group participants also explained that it allowed them to “wait and see” if their contribution was necessary or if it was possible to fill in gaps in responses as opposed to having each scholar produce a comprehensive individual response. Qualitative data also highlight the interactive benefit of the platform because the discussion forum allowed researchers to cooperate on a final response and mitigated burden of a comprehensive response from individual respondents that other-wise occurred via emails. Participants also reported the platform to be less individualized than emails, leading them to not regularly log on to the platform unprompted. This revealed that it was necessary for the RPC implementation team to embed the platform into user routines, including email correspondence, and that of the RPC implementation team’s routine of using the platform for managing Rapid Response activities.

Study participants expressed limited time for venturing into the platform for more casual perusing of policy opportunities, which was consistent with observational data. Specifically, topic-specific communities were developed as an open forum for researchers to build community within respective disciplines, but were rarely used independently by participants. Routines, or a lack thereof, might explain the selective use of the policy tools platform feature. If researchers are not routinely in need of policy information, they are unlikely to use those features. Nevertheless, the platform provided a consistent and centralized location for participating researchers to find information, trainings, and tools for their policy engagement. Focus group participants also reported using the platform content and rapid response experiences in teaching activities for training students on policy engagement strategies.

The platform featured a recognition system consistent with gamification theory, which we hypothesized would provide positive reinforcement associated with increases in policy engagement behaviors. This recognition system included badges that were visible on individuals’ platform profiles and were automatically generated using platform metrics of engagement. The results of this field experiment revealed that the gamification feature was not supportive of researchers’ rapid response contributions, and if anything, the feature was received poorly (Appendix 3). A focus group revealed that many participants were largely unaware of the badge feature, were perturbed by frequent emails (e.g., which initially included badge recognition), and advised that platform badges did not feel particularly rewarding (Appendix 1). Instead, participants advised that the RPC implementation team consider recognitions that would be more consistent with academic norms for listing accomplishments on curriculum vitas (CVs). Consistent with design thinking, we used this qualitative feedback to reformulate and test an improved feature – an award for policy engagement coupled with a letter of recognition and exclusive access to resources in the platform that promised to boost their accomplishments in their CVs. Instead of waiting to examine the long-term effectiveness of the award on rapid response contributions, the study team designed a message test demonstrating that the click rates on the coordinating group’s social media pages were higher in the award condition (Appendix 4). This provided the implementation team with the confidence to roll out the award system to other participants. Focus group feedback was also positive and highlighted that the recognition among upper-level staff at academic institutions was used as reasoning for covering travel to RPC Congressional events (Appendix 1). Feedback also revealed that the emailed award was sometimes not seen by participants, underscoring the burden of email for scholars, and prompted the investigative team to send nudges to future awardees to ensure receipt.

### Leadership communication

Alongside the platform testing and refinement, the study team routinely gathered experimental data to test hypotheses of leadership communication. Our leading hypothesis involved articulating the benefits of engagement and mitigating perceived barriers (e.g., time). Thus, we tested various messaging strategies and the results ultimately showed that researchers were not more likely to engage when reminded of the benefits of their participation (Appendix 5–7). Overall, persuasive tactics backfired. All of these attempts were unsuccessful in significantly changing enrollment rates, suggesting that messaging motivational factors or downplaying barriers was not effective for persuading scholarly policy engagement. If anything, these message strategies backfired, perhaps because they seemed inauthentic or sparked reactance. Messages that sound the most natural were received most positively. Comparable to tests showing that persuasive, motivational messages were ineffective for prompting network enrollment, researchers were also not persuaded to contribute to rapid responses designed to convey benefits, but were actually more likely to engage in response to the control message (Appendix 8).

Instead of persuasion tactics, study findings suggest that leadership communication might be better defined in relational terms that build connections between participants and with leadership. Messages that conveyed authentic appreciation and enthusiasm, were humanizing or empathetic, or cultivated connections between scholars were largely found to be effective for supporting researchers’ policy engagement. Specifically, we learned it was not beneficial to prospective enrollees to offer a time to talk about the RPC in a meeting (Appendix 9) nor to personalize emails to the recipient (Appendix 10). Instead, we found that there was a human and social element relevant to researchers’ decision to enroll. Emails that contained a narrative about why a team member found this work to be meaningful outperformed the standard recruitment language (Appendix 11). A similar test deployed to the network introduced new implementation staff members and upheld the importance of communicating with narratives/values (Appendix 12). Messages that conveyed enthusiasm (e.g., “revolutionary”; Appendix 13), empathy (e.g., “we’re hoping it’s manageable with your other commitments”; Appendix 14), niceties (e.g.,” Hope your week is going well,”; Appendix 8), and creating modest goals (e.g., “anything you can do helps”; Appendix 8) also appeared to encourage engagement. We also found that it was not always easy to strike the right balance of personable and authentic messages, since one of our attempts to create a personal narrative about our hopes for making it easier for them to engage failed to increase engagement (Appendix 15). However, while we found consistent evidence that researchers were most compelled to enroll in the RPC when emailed by colleagues in their respective fields as compared to emails received by the RPC implementation team (Appendix 16), we did not observe this difference when a staff member mentioned the name of a mutual colleague who recommended their involvement (Appendix 16). Overall, these results demonstrate the importance of interpersonal relationships between RPC implementation staff and leadership with the research community for facilitating collective action.

Additional tests of leadership communication relate to the need for creating a rewarding experience consistent with academic norms. We found that a message using the word “recognition” was clicked 6x more than its comparison (Appendix 17), whereas emphasizing nonacademic benefits (e.g., science communication skills) was ineffective and appeared to backfire (Appendix 8). We also found that it can be difficult to strike a balance of conveying empathy of challenging situations (e.g., “tough to balance”; Appendix 14) as compared to over-emphasizing real barriers faced by academics (e.g., “account creation is annoying”; Appendix 13). Not only were the enthusiastic vision-oriented messages particularly successful, we also found that messages that underscored the team’s policy impact were more successful for promoting network enrollment as compared to an emphasis on academic activities (e.g., articles and grants; Appendix 18). Together, findings suggest that researchers may be more likely to invest when authentically inspired, such as when they believe they can effectively accomplish social impact. Thus, we define leadership communication as cultivating trust in both the vision and the collective action process. Since transparency regarding the ongoing policy initiatives should also correspond with this notion and the broader literature, we also tested a process of sending updates of the final evidence synthesis to participants. Unfortunately, we did not see an effect on researcher engagement (Appendix 19) and this hypothesis was also not supported with message testing (Appendix 20). A focus group revealed yet again that researchers were largely unaware of these attempts because they do not see all emails and find additional emails that intended to convey transparency as burdensome (Appendix 1); thus, we adapted our transparency approach by posting updates on the final product and meeting outcomes within the platform.

## Discussion

Decades of leadership offered by Dante Cicchetti have elevated developmental science in ways that recognize (1) the interplay between normal and abnormal developmental processes in the emergence of psychopathology, (2) the multiple levels of functioning (from the cellular to the societal) at which mechanisms of action can be intervened upon, (3) that the organization of behavior unfolds over time and thus sensitive periods and contexts matter, (4) that deleterious consequences of early adversity and abuse are far from inevitable, and (5) that developmental context and timing matter. This elevation has culminated in a collective of once-disparate and siloed fields into a coherent discipline of Developmental Psychopathology (Cicchetti & Toth, 2009), bringing together transdisciplinary researchers who leverage an essential scientific framework to produce sound and valid inquiries into the human condition. Cicchetti’s devotion to elevating Development and Psychopathology as a distinct and identifiable discipline has resulted in a means for scientists to work toward collective impact and a trusted source for evidence-based policymaking.

The RPC model is an infrastructure that has been designed to support policymakers’ use of evidence by connecting them with researchers and by aiding researchers in the translation of disciplinary research. By further brokering the relationship between policymakers and researchers, the RPC helps create trusted pathways through which sustained contact can be realized such that policymakers seek out researchers who represent an identified discipline whenever they need the traction of cutting-edge research to support policy reform. Likewise, researchers can rapidly respond to legislative requests with succinct, erudite substantive products that can be readily integrated into persuasive language for supportive materials such as policy briefs and factsheets, but also integrated into legislative language and action.

Although the RPC has been empirically studied and shown to be effective at improving policymakers use of evidence in policymaking and improved researchers’ ability to translate science into policy-relevant messaging, the RPC infrastructure needed optimization that increased researcher engagement. The rapid trials reported in this paper were designed to identify processes that could be improved for optimizing researchers’ experiences participating in the RPC model’s RRN. The primary lesson learned involves the value of leveraging digital infrastructure to improve communications. This work presents the first experimental evidence of virtual tools for organizing the policy engagement of researchers.

Our study findings suggest little benefit of persuasive messages encouraging researchers to engage in policy efforts. In fact, marketing-type methods could be detrimental to policy engagement initiatives. Instead, we find that researchers are motivated by genuine enthusiasm for achieving social impact, and rapport building efforts that develop connections with and among scholars may be most effective. They also value recognition of these efforts so long as it complies with the incentive structures of academia. While a virtual policy network provides some self-reported benefits, there is more work to do to test how this enhancement supports scholars, and which scholars, in ways that were not measured in this study. Although there is no singular strategy for motivating researchers’ policy engagement, these findings align with the theory that authentic relationships are most likely to achieve our collaborative goals.

The vision for the new and improved RRN involves several practices for revolutionizing a digital infrastructure for collective action in a scientific discipline. First, communications about essential policy opportunities will be streamlined in the virtual platform by using it to post policy opportunities, emailing select researchers (who have relevant expertise) to direct them to the post, and providing updates for transparency by replying on an existing thread rather than creating a new standalone email. This can reduce the frequency of email communications with participants to an essential basis and allows researchers to add to existing replies instead of creating a new unique reply. Importantly, this feature also streamlines policy associate time by reducing the time it takes to reconcile contributions by reducing redundancy among respondents. The virtual platform can also centralize other offerings for eager participants to engage in trainings, networking opportunities, review current policy efforts in their study area, and learn about policy-relevant research in domains outside their areas of expertise.

When the RPC or its features are articulated to researchers, leadership communication strategies involve highlighting the motivating vision for research impact, empathizing or aligning with realistic academic norms and pressures, showing authentic appreciation and enthusiasm, and developing interpersonal connections with or between participants. For instance, future recruitment efforts will enlist scholars within the discipline to support the outreach effort since this within-discipline was seen as so effective for engaging their peers. Recognizing the importance of appropriate recognition and reward, the optimized RPC also systematizes the Policy Scholars Award that recognizes time and effort for policy contributions and embeds this recognition within the platform badges feature. Those receiving the award receive access to additional resources such as a policy-oriented CV template.

In sum, these study findings contribute to the limited literature examining infrastructure for facilitating researchers’ policy engagement. While the study strengthens what is known about supporting child and family researchers’ policy engagement, the study is not explicitly intended to generalize to vastly different areas of study. Although the study is strengthened by the use of observational metrics in experimental trials, the study metrics do not always detect less overt forms of engagement. For instance, the metrics regarding participants’ contributions do not reflect the access and viewing of the platform, thus this null finding does not capture the contemplative stage of viewing the opportunity without responding. Coupled with focus group findings, scholars’ engagement with the platform may be underestimated since they only choose to contribute when there is a gap in responses from their colleagues. Such “lurking” behavior may actually reduce the ability to detect an effect of the platform on engagement rates. Future research could seek to capture earlier indicators of engagement, such as using click rates (e.g., email-only participants could click on a google doc). Another challenge inherent in this study is that language is interpretive, which makes it difficult to consistently operationalize constructs in message tests (i.e., leadership communication). This challenge is primarily addressed by repeatedly testing the same construct in various contexts to examine when the findings converge, diverge, and why that might be the case (e.g., variation in the messages). Thus, the number of trials and focus group findings presented in this paper should be viewed as a strength since it provides a fuller picture regarding the experiences of scholars engaging in a virtual policy network.

### Future directions for the field

The evolving discipline of Developmental Psychopathology underscores the imperative of not only considering policy implications in our research, which infers a need for supporting research translation and policy engagement. This integration is not purely theoretical but represents a practical need for sustaining researchers’ ability for sustained engagement in the context of structural challenges that hinder their policy efforts. The interplay between individual development and societal factors necessitates a holistic approach – one increasingly inclusive of public policy – and one that addresses the research system.

### Prioritizing policy integration in research

Building upon our experimentation and empirical work, it becomes evident that policy-relevant research can have not only actionable implications, but it can also be part of the scientific inquiry itself. Researchers are uniquely positioned to inform policy through evidence-based approaches. This requires a conscious shift towards policy-relevant research questions and design. We give greater meaning to our work when we avoid conducting our research in isolation – instead, envisioning how these findings can be translated into actionable policies. Collaborations between researchers and policymakers and the communities we both serve are crucial to success in this area, as they bridge the gap between theoretical knowledge and practical implementation. As we look to the future, considering the policy implications of our work should start early in study development and be a frequent topic of discussion throughout the study process. Below weidentifykey future directions for the field.

### Enhancing researcher-policymaker collaboration

To facilitate this collaboration, strategies must be devised for improving communication and understanding between researchers and policymakers. This involves not only speaking a common language, but also appreciating the constraints and incentives of each community. Learning from successful collaborations can provide valuable insights. These partnerships must go beyond adhoc interactions, aiming instead for sustained engagement and mutual learning.

One powerful example of a successful collaboration and a tangible example of the RPC model’s effectiveness further illustrates the power of researcher engagement in supporting policymaking. As part of the NICHD P50 Capstone Center of Excellence, The Translational Center for Child Maltreatment Studies (TCCMS; P50 HD089922; PD Noll), the RPC was adopted for specific use in the advancement of legislative priorities geared toward sound child welfare practice issues and increasing public investment in child maltreatment prevention and treatment. By cultivating an RRN in this substantive area, the P50 mechanism allowed the research team to use the RPC model to develop legislative partnerships related to the Reauthorization of the Child Abuse Prevention and Treatment Act. First, an RPC policy associate who worked directly with the TCCMS met with legislative staff in key Committee offices charged with amending the law. These meetings involved conducting a needs assessment to understand what goals policymakers had related to the bill, and then following up on these interests by mobilizing rapid responses from researchers participating in the RPC RRN. This responsiveness not only demonstrated the value of research related to their interests, but supported rapport development that was critical for the TCCMS to becoming a trusted source of information. Responses included a policy brief authored by TCCMS researchers and subject matter experts from the RRN. Then, two briefings were hosted in both the House and the Senate in which TCCMS researchers spoke and key Committee staffers attended, including those who later approached these researchers with an invitation to testify in front of the US House Committee on Education and Labor’s subcommittee on Civil Rights and Human Services. It is not typical that researchers are asked to testify at hearings like this unless trust and rapport have been previously established. The TCCMS then formed a written response for the official record that summarized extant research regarding the costs of child abuse and neglect, as well as the impact and effectiveness of prevention programs. A trained RPC policy associate coached and prepared the TCCMS scholar to deliver oral testimony and field questions. After the hearing, the TCCMS was asked by the Senate Health, Education, Labor, and Pensions committee to review the proposed legislation and offer educational comments on the related research evidence. CAPTA passed the House in May 2019 with a historic appropriation increase in state prevention program funding. This example illustrates the importance of rapport building strategies for becoming a trusted source of information in a domain that is relevant for policymakers’ current legislative goals.

### Training and education for policy engagement

Similar to other developmental processes, the foundation for policy engagement needs to be laid early in a researcher’s career. This necessitates an evaluation of current training and education programs in Developmental Psychopathology. Academic institutions must incorporate curriculum that not only teaches research methodology, but also imparts skills in policy engagement and translation of research. Mentorship programs can play a pivotal role here (e.g., Society for Research on Child Development, Research-to-Policy Fellowships) by linking junior researchers with experienced policy-engaged scholars. These training opportunities focus on providing experiential learning opportunities that build scholars skills around engaging with policy communities. In some cases, they can catalyze experiences that orient scholars toward thinking about policymaker needs as they develop their own programs of research.

### Technological advancements and research dissemination

New technologies, such as the RRN’s virtual policy network for research translation, hold tremendous potential in the realm of research dissemination and policy impact. Digital platforms such as social media can amplify the reach of research findings; however, it is crucial that strategies are guided by a robust understanding of intended impact, purpose, and audience. Communicating into the abyss, writing briefs that rest on a dusty shelf (i.e., never read by policymakers), or creating an echo chamber among colleagues may not align with intended effect. Strategic policy initiatives identify an intended target audience and then leverage the appropriate communication channels in which that audience may be reached. The SciComm Optimizer for Policy Engagement is an infrastructure that parallels the RPC as it has been used to amplify the products scholars produce in the RRN by increasing their reach and visibility with state legislators via email (Scott et al., 2023). Digital technologies also bring challenges and ethical considerations, such as ensuring the accuracy of disseminated information, appropriate use of artificial intelligence, and combatting misinformation.

### Institutional support and incentives

The importance of academic institutions in nurturing policy-engaged research cannot be overstated. Broadly speaking, the scientific institutions are interconnected with both producers of research (e.g., universities) and funders of research (e.g., National Institutes). Institutional support can be a catalyst for significant advancements. Just as these interventions require a supportive infrastructure to thrive, so too does policy-relevant research necessitate an institutional ecosystem that not only supports but actively incentivizes such endeavors. This involves recognizing and rewarding the efforts of researchers who engage in policy-relevant work. Institutions must strike a balance, ensuring that academic rigor and policy engagement are not seen as mutually exclusive but as complementary and mutually reinforcing pursuits.

Addressing these barriers will be essential as they often manifest as institutional inertia, lack of funding, or insufficient interdisciplinary collaboration. Just as systemic factors play a critical role in shaping individual developmental outcomes, they also significantly impact the ease with which researchers can engage in policy-related work. Further, these systemic barriers often manifest disproportionately for historically marginalized communities – requiring special attention to issues of equity and inclusion. Recommendations for structural changes within academia should be grounded in a nuanced understanding of these systemic challenges, aiming to facilitate a more conducive environment for policy-focused research.

One of the biggest hurdles policy-engaged researchers face is working for an academic institution that restricts them from engaging with policy communities. Providing education and research on a particular public problem to elected officials is a fundamental role of the scientific community – and should not be conflated with lobbying. The federal definition of lobbying is clear that it constitutes the practice of asking elected officials to take a specific position on a specific piece of legislation. Education and engagement that researchers can do otherwise, without lobbying for partisan solutions, has the potential to align with the service missions of our scientific institutions and elevate the public benefit of research knowledge.

### A call to action

In reflection of Dante Cicchetti’s legacy and consideration of the opportunities for new frontiers of study, this manuscript has attempted to chart a course for the future of the discipline, emphasizing the critical importance of policy engagement and collaboration. The discipline stands at a crossroads, where the potential for societal impact is immense, provided researchers, policymakers, and institutions work collectively towards shared goals. This requires a concerted effort to embrace the challenges and opportunities presented by an increasingly complex world.

We end this tribute by offering several tangible activities that we suggest to researchers who wish to enhance the impact and policy-relevance of their work and the research that is shaping the discipline of Developmental Psychopathology. Beyond open opportunities for participating in the RPC (TrestleLink, n.d.), Table 1 outlines action steps and implementation strategies for a host of activities that can orient researchers toward policy-relevant scholarship and engagement. These recommendations are meant to provide a foundation of knowledge and suggestions for a collective call to action that is becoming clear: Developmental Psychopathology as a discipline must not only advance our understanding of human development but also actively contribute to informing the policies and practices that impact the human condition across the lifespan.

## Supplementary Material

Appendix file

## Figures and Tables

**Table 1.** Future opportunities for policy-relevant scholarship and engagement

Action Step	Description	Implementation Strategy
1. Prioritize Policy-Relevant Research Questions	Focus on research questions that have clear implications for policy development. Interdisciplinary research can address complex policy issues from a holistic perspective.	Regularly review current policies and societal challenges to align research questions with real-world needs. Collaborate with researchers in other departments to include economic, law, public health, and sociological perspectives.
2. Foster Collaborations with Policymakers	Establish and maintain relationships with policymakers to facilitate the translation of research into practice.	Attend policy forums, participate in governmental committees, and actively seek opportunities to collaborate with policy organizations.
3. Improve Communication Skills	Enhance skills for effective communication with nonacademic audiences, particularly policymakers.	Attend workshops on science communication, practice presenting research in layman’s terms, and use various media platforms to disseminate research findings.
4. Advocate for Policy Engagement in Curriculum	Encourage academic institutions to include policy engagement and translation in their curriculum.	Propose new courses or workshops focused on policy engagement and collaborate with faculty from policy studies to develop interdisciplinary programs.
5. Utilize Technology for Dissemination	Identify a target audience of research communications and then leverage digital platforms that reach that audience.	The SciComm Optimizer for Policy Engagement is one model for directly reaching policymakers. Social media platforms may increase visibility and impact among a public audience or among your colleagues, depending on your followers.
6. Seek Institutional Support	Advocate for institutional recognition and support for policyengaged research.	Propose institutional policies or incentives for policy-engaged research. Highlight the benefits of policy engagement in terms of societal impact and funding opportunities.
7. Address Systemic Barriers	Identify and work towards overcoming systemic barriers to policy engagement within the academic and research environment.	Conduct surveys or focus groups to identify specific barriers at the institutional level. Advocate for changes in academic policies or structures that inhibit policy engagement.
8. Stay Informed about Policy Developments	Keep abreast of current and emerging policies related to Developmental Psychopathology.	Subscribe to policy newsletters, attend policy briefings, and participate in policy-focused academic conferences.
9. Mentorship in Policy Engagement	Engage in mentorship programs where experienced policy-engaged scholars guide junior researchers.	Establish or participate in mentorship programs focused on policy engagement within academic institutions or professional organizations.
10. Advocate for Structural Changes	Advocate for structural changes within academia to facilitate policy-focused research, such as funding policies that favor policy-relevant projects.	Participate in academic governance, committees, or advocacy groups to push for reforms that support policy engagement.
11. Pursue Policy-Oriented Funding Opportunities	Actively seek and apply for grants or funding opportunities that prioritize research with policy implications.	Regularly search for and apply to funding opportunities specifically aimed at policy-relevant research. Collaborate with grant writers or funding experts to craft compelling applications.
12. Conduct Policy- Oriented Research Workshops	Organize and participate in workshops aimed at discussing and promoting research with direct policy applications.	Collaborate with research institutions and intermediaries to organize workshops. Invite policymakers to discuss the practical implications of research findings.
13. Develop Policy-Briefs and Reports	Translate research findings into policy briefs and reports that are accessible to policymakers and the general public.	Collaborate with communication specialists to create concise, impactful policy briefs and reports. Distribute these through institutional channels, at conferences, and via online platforms.
14. Regular Policy Feedback Mechanisms	Establish regular mechanisms for feedback from policy communities to ensure that research remains relevant and impactful.	Set up advisory panels or forums with policymakers and stakeholders to provide regular feedback on research projects.
