# Supplementary material for: Shifting the paradigm of research-to-policy impact: Infrastructure for improving researcher engagement and collective action
Source: Dev Psychopathol. Author manuscript; Available in PMC 2025 Jun 1. (PMC11416572; doi:10.1017/S0954579424000270)
Supplement: Appendix file [file NIHMS1989498-supplement-Appendix_file.docx]

***Table S.1. Summary of Methodological Approaches***

| **Method** | **Purpose** | **Frequency** | **Example** | **Metric** |
| --- | --- | --- | --- | --- |
| Simultaneous Message | Bulk message testing /  Simplest to deploy | Once | Narrative messages for researcher enrollment) | Opens/Clicks, obtained in a Client Relationship Management software; participation or enrollment records |
| Repeated Message | Rapid Response messages /  Minimally-disruptive | Multiple; until n=500 | Explain benefits of participation in an RR | Responses, coded by study team members |
| Structural | Process or feature testing | Once | Gamification features | Responses; clicks |
| Focus Group | Understand platform experiences | 4 / year | Feedback on gamification | Transcript coding |

***Table S.2. Summary of Trials Conducted***

| **Appendix Number** | **Study Construct** | **Sample Size** | **Method** | **Metric** | **Result (Significant or not)** |
| --- | --- | --- | --- | --- | --- |
| 2 | Platform engagement | 849 | Simultaneous | Clicks/opens/enrollment rate/ RR contributions | Not Significant |
| 3 | Gamification | 694 | Structural | Clicks/opens/enrollment rate/ RR contribution | Not significant |
| 4 | Policy Scholars Award | 76 | Structural | Clicks/opens | Significant |
| 5 | Intrinsic vs Extrinsic Motivation | 781 | Simultaneous | Clicks/opens/enrollment rate | Not significant |
| 6 | Recruitment 4 - time | 1,435 | Simultaneous | Clicks/opens/enrollment rate | Not significant |
| 7 | RR trial 4 - urgency | 184 | Simultaneous | RR contribution | Not significant |
| 8 | Emphasizing benefits backfired | 164 | Repeated Message | Clicks/opens, RR contribution | Significant |
| 9 | Recruitment - meeting offer | 879 | Simultaneous | Clicks/opens, completion of form | Not Significant |
| 10 | Personalizing | 288 | Repeated Message | Clicks/opens, RR contribution | Not significant |
| 11 | Recruitment - personal narrative | 1,689 | Simultaneous | Clicks/opens | Significant (partial) |
| 12 | PA intro trial | 470 | Simultaneous | Clicks/opens | Significant (partial) |
| 13 | Platform launch email | 446 | Simultaneous | Clicks/opens, enrollment rate | Significant |
| 14 | RR trial 3 - empathy | 247 | Repeated Message | Clicks/opens, RR contribution | Significant |
| 15 | Launch event RSVP | 432 | Simultaneous | Clicks/opens, form completion/registration | Trending but not significant |
| 16 | Recruitments - warm hand-off | 123, 116, 94, 202 | 4 Simultaneous Tests | Clicks/opens, form completion | Significant (partial) |
| 17 | Gamification nudge | 151 | Simultaneous | Clicks/opens | Significant (partial) |
| 18 | Recruitment 3 – credibility types | 1643 | Simultaneous | Clicks/opens, enrollment | Significant (partial) |
| 19 | Transparency structural | 512 | Structural | RR contributions | Not significant |
| 20 | Transparency MT | 524 | Simultaneous | Clicks/opens | Not significant |

**Appendix 1: Focus group Report**

***Methods Summary***

| **FG 1 Topic** | **Platform and Transparency**: To get a general understanding of participants’ familiarity with the RPC’s virtual policy engagement platform and what their experience has been like. Also to understand participant experiences of receiving debrief emails that attempted to increase transparency about the result of their participation. | | | |
| --- | --- | --- | --- | --- |
| **FG 1 Sample** | *N* = 6. Participants included scholars enrolled in the virtual policy engagement platform (Appendix 2) and received the transparency debrief messages (Appendix 19). 50% of participants had not engaged much with the platform, while 50% engaged at least a little. Participants were selected to be diverse across race and gender. | | | |
| **FG 1 Primary questions** | 1. In general, how has your experience with the platform been so far? 2. Which aspect or feature of the virtual policy engagement platform has been most helpful or useful to you when engaging with the Research-to-Policy Collaboration? 3. Is there an aspect or feature of platform that you either do not like or have found difficult to use? If so, please describe it. 4. Have you experienced any barriers to accessing or using the virtual policy engagement platform? If so, what are the barriers that you have experienced? 5. How could the virtual policy engagement platform be improved? In other words, what are your suggestions for how to make the virtual policy engagement platform be more accessible or easier to engage with, or more supportive of your research translation efforts? 6. What are your thoughts about the emails you receive from RPC? 7. What are your general thoughts and/or reactions to the debrief emails? (i.e., transparency intervention) 8. We saw that when people received debrief messages, they were sometimes less likely to engage subsequently. Why else do you think that might be? 9. What were your perceptions about synthesis products that were created? 10. How/when would you prefer to receive such information, if at all? | | | |
| **FG 2 Topic** | | **Gamification**: To discuss the badge feature on the RPC Virtual Engagement platform (Appendix 3) to better understand researchers’ impressions of it and how useful (or not) it is. | | |
| **FG 2 Sample** | | *N* = 7. Participants included scholars that were included in the gamification condition. Participants were selected to be diverse across race and gender. | | |
| **FG 2 Primary questions** | | 1. Can you please raise your hand if you are aware of the badges feature in the platform? 2. If yes: Raise your hand if you feel you have a good understanding of how to work towards and earn badges. 3. For those who were aware of the badges: how do the badges impact your activities in the platform? For those who just found out about them, how do you think they will impact your future activities in the platform? 4. What is your perception of the purpose or value of badges? 5. What could make the badges feature more appealing or rewarding? 6. Probe: What types of activities or tasks would you like badges to trabe connected to? 7. Probe: What are the best ways to recognize your efforts? 8. What other features, if any, would you like to see in the platform? | | |
| **FG 3 Topic** | | | **Award:** To hear about participants’ experiences with and impressions of the RPC Scholars Award. Participants received an email notifying them of the achievement, with a corresponding package of access to an online community, a letter of recognition, and a template for reporting activity with the RPC on their CV. | |
| **Sample** | | | *N* = 8. Participants included scholars who received the RPC Scholars Award (Appendix 4). Participants were selected to be diverse across disciplines, faculty positions, engagement levels with the RPC, race, and gender. | |
| **FG 3 Primary questions** | | | 1. First, can you please raise your hand if you were aware of this achievement? 2. Overall, what are your impressions of this package? 3. Do you find the award offerings (community, letter, and template) to be meaningful? 4. Does the award feel appropriate for the degree of effort needed to achieve it? 5. For those of you who were aware of the award and offerings, how do you feel like it affected your perception of and work with the RPC? Or, for those unaware, how do you expect it would affect your perception and work? 6. Probe: Do you feel more engaged? Less engaged? No change? 7. Is there anything you think would improve this award? 8. Probe: How would you have preferred to receive notification of this award? 9. Probe: What would you like to see in the online community? | |
| **FG 4 Topic** | | | | **Policy Tools:** To discuss the policy tools aspect of the RPC Virtual Engagement platform to better understand researchers’ impressions of it and how useful (or not) it is. |
| **FG 4 Sample** | | | | *N* = 6. Participants included scholars that were included in the policy tools page condition. Participants were selected to be diverse across engagement levels with the RPC, race, and gender. |
| **FG 4 Primary questions** | | | | 1. Can you please raise your hand if you are aware of the Policy Tools page? 2. If yes:  Have you navigated to the Policy Tools page? Which tool have you used, if any? 3. How helpful was the tool? How did you use the information from the tool? 4. If no: Which tools were you most excited to see on the page? 5. How do you feel about having access to the Policy Tools page? 6. Probe: is access to this page something that is valuable, motivating, or empowering to you? 7. How could the RPC improve the Policy Tools? 8. Probe: to make them more useful, relevant, or helpful to you 9. Probe: what are the things you struggle with in order to effectively do policy work? 10. What tools, if any, do you wish you had access to? What is the “ideal” tool that would help with your policy engagement? |

***Content Analysis Thematic Report***

1. **Barriers to using the platform:** Researchers named several barriers that resulted in decreased engagement in the virtual platform and the RPC process.
   1. *Email is a burden:* Perhaps the most reported barrier for engaging with the platform was being overburdened by emails, which is a common method of communication for those employed in the academic sector. For example, “Related to emails, I think I was getting individual emails before the new online platform. And then suddenly the online platform started, and I got a barrage of emails that was overwhelming. And so, I quickly went in and set it to mute, or whatever, because I couldn't deal with that level frequency of emails. And so because of that I haven't been getting the email, which is a welcomed break, but also means that I haven't been interacting with the platform” (Platform focus group). Researchers also stated that some emails did not offer an incentive to participate. For example, “You would have to give me a reason why I would want to do that. And you know, I mean that because this I'm sure, everyone gets a lot of emails and solicitations on a regular basis. And so, they’re just there. I think it just needs to be a clear through line about what the people's benefits are for learning this additional system and engaging with it on a regular basis and emails you know” (Gamification focus group).
   2. *Transition from email to the RPC platform:* Researchers stated that the transition from email to the platform resulted in rapid response solicitations not being as individualized. For example, “There's one thing that made that felt different about getting the email from you all, and when getting the email with the request, it kind of made me feel like, oh, they like, really need my help. I'm an expert, and it's like I need to help with this, or else it like won't, maybe like it can't, move forward. But then, as part of this platform where we're all connected, one of the weird downsides I realized, in fact, it's not only me who can help. There are many of us, but something like knowing that the research request can be answered by actually a lot of people makes me a little less motivated” (Gamification focus group). Researchers also stated that learning how to navigate the platform reaped more burden than benefit in some cases. For example, “It was just too much, and I couldn't put in the time to figure out even what the point of the platform was. I didn't see what the payoff would be of putting in the time to figure all this out” (Policy tools focus group). Another researcher agreed, “I went on it when I first logged on, and you know, honestly, I guess I found it a little overwhelming that there was just it felt like there was a lot of material that would have to be sifted through before I could potentially identify something that I might want to get involved in” (Policy tools focus group).
2. **Researchers’ views regarding collaboration opportunities offered by the platform:**
   1. *Researcher Collaboration:* Researchers echoed that the collaborative nature of the platform was useful for gauging how to respond to rapid response solicitations. They also stated that it would be useful to have Congressional offices included in direct feedback loops for their responses. For example, “I think it's nice to get to see other people who want to contribute to this area. And also I don't know how often it's used by legislative offices that it could be that's kind of a starting point for them … it's quite user-friendly” (Platform focus group). “I feel like sometimes it's hard to judge how helpful the responses are because you don't always hear anything back from the staffers or the lawmakers. So sometimes I post responses, but I have no idea if it's actually helpful or not. So I think that that this award was a little bit helpful in that OK, maybe those responses are worthwhile” (RPC policy scholars award focus group). A researcher also expressed that they appreciated being able to vote on promoting other researchers’ discussion posts, “You know I like it as a tool, for you know, getting some folks together to, you know, pull some research. I like the little voting system so that I can know if folks think I've gone off the deep end or not, right?” (Platform focus group).
3. **Researchers’ perceptions towards platform resources:** Researchers were largely unaware of the different resources available on the platform. Across focus groups, researchers recognized the usefulness of the different resources but were not inclined to use them outside of their regular duties on the platform.
   1. *Badge function:* Overwhelmingly, researchers stated that the badge function did not align with researchers’ professional goals. For example, “For me, these badges don't connect closely enough to my own goals” (Gamification focus group). Instead, it was suggested that the badge function be used to reinforce behavior that would normally occur and fit into researchers’ busy schedules. For example, “I don't know if I'm trying to sign myself or anyone up for extra work to get badges, but those kinds of things that I would naturally do to get that kind of recognition I think I would make me more inclined to participate” (Gamification focus group). Regular platform behavior would include responding to rapid response solicitations, which researchers thought would be a useful badge to receive, “I can't recall if this is a badge or not, the one where you showcase, whether or not you've contributed to one of the products that you send out when appropriate... the rapid response” (Gamification focus group).
   2. *Policy tools page:* Researchers stated that while the policy tools page could be useful to their future work, it was unlikely that they would actually use the page. Some researchers found that the page was intimidating. For example, “Another thought that I had is while these tools are nice, I wonder how realistic it is for someone to just pop on and feel completely confident and use them independently. And this is just from my own past experience and trying to do this work. It can be intimidating. It can seem like it'll be ineffective, and it's just helpful to be coached on some of this stuff” (Policy tools focus group). Researchers found that the page could be used for shaping their own research. For example, “I think it's great for some to help with preliminary research,” “I could just use this in my own research truthfully forget about outreach. But I mean, I study public policy, issue attention, and agenda setting” (Policy tools focus group).
   3. *RPC policy scholar award:* Researchers expressed that the RPC policy scholar award benefited their professional goals in their institutions, leading to increased engagement in the future. For example, “I would reiterate the importance of it because academia awards these kinds of forms of recognition, especially when you're on the tenure track, it is really helpful for them to see the value of translating research to policy. And so, the more that, you know, my department head can say, oh, look, one of my faculty members got this award. The Dean is pleased, and you know, it's just sort of the, the structures of academia appreciate those things, even if sometimes that's not the motivation” (RPC policy scholars award focus group). Another example included, “I-I didn't think much of it, but I mentioned it to our Director of Research and in this space we're just desperate for recognition of any kind in the school but for individuals as well. And it got some attention to uh my surprise and you know, I don't um, sometimes it's hard to toot your own horn because you focus so much on the stuff you're not getting done. But this one, this one helped. So let me just extend my gratitude for that. I think I'm probably a little bit more engaged” (RPC policy scholars award focus group).
   4. *A tool for education:* Researchers reported that the RPC model was worth educating their colleagues and students about. For example, “I teach policy analysis in a college of medicine, and I navigate the platform when I teach my course, especially the rapid responses I want to show students recent examples of policy analysis and depending on the topic I just navigate the website and present some of those examples. Every spring, and I think I use four or five of your summaries in my last semester. So that's my usage with the platform. And for my own use, I think is, friendly” (Platform focus group).
4. **Overall Appreciations for the RPC:** Researchers consistently appreciated the RPC for their role in the translation process from research to policy. They also found the platform itself to be intuitive and a supportive tool for engaging with the RPC.
   1. *Appreciation for the platform:* Researchers expressed that they appreciated the RPC platform’s ability to foster collaboration. For example, “I think that one of the things that RPC does really well is fostering collaboration. And you know I've never really worked on anything alone, you know it's been like this meeting of the minds, particularly the policy briefs so that does take some of the pressure off, knowing that, you know, I don't have to figure out everything but kind of piggy backing off of one another. And you know, focusing on our areas of expertise and kind of bringing that all together. And I think that's something that the website can possibly facilitate” (Policy tools focus group). Researchers also expressed that they valued opportunities to help the platform improve. For example, “I want to commend you all also because this new platform is amazing you know, for all like we're talking about how to optimize it and our use of it” (Gamification focus group).
   2. *Appreciation for bridging the gap:* Researchers expressed how the RPC bridged the gap between communicating research to policy makers, which often is not a role undertaken by those employed in academic institutions. For example, “It sort of indicates I think the goal of the RPC to continue and expand its efforts, which I do appreciate because we don't always make the connections with the policymakers who are interested in our particular areas of expertise” (RPC policy scholars award focus group). Another example included the barrier of getting research outside the ivory tower of academia, “Whereas I've heard in universities, it can be hard to get your work sometimes out of the ivory tower, even if you understand that it's very important and could have important implications for the real world” (RPC policy scholars award focus group). Researchers also articulated the importance of this work for breaking down structural barriers such as subject areas being siloed from each other. For example, “I mean I think what's nice about the platform is that there's some space for us to kind of break down barriers between like you know, siloed fields of study and a lot of times actually I think that's what policymakers need to be hearing about so even though my area is actually in education, like there's intersections with health, there's intersections with social work, there's, you know, so, but often times we're very siloed” (RPC policy scholar focus group).
   3. *Recognizing researchers’ participation:* Researchers extended gratitude for the RPC recognizing their participation in the network. One example of this recognition included the RPC policy scholar award. For example, one researcher shared how the award helped fund their travel to Washington DC to participate in a Hill Day, “The award also enabled my college to see the value in what I was doing. I think they did understand the value of it, but I think it was just another piece of value that they saw. I didn't really have to ask very much for them to fund me to go to DC. They did so willingly after I received the award, fully funded me. So I think that that also helped um legitimize some things for promotion and tenure criteria because we rewrote that this summer as well. Um and so working with policy collaboratively is now a different category. And so I think all of those things really kind of helped further that a little bit, not just for me personally, but also in my institution” (RPC Policy scholars award focus group).

**Appendix 2: Platform Engagement**

**Method:**

A sample of 849 researchers who had enrolled in the Rapid Response Network had been randomized to one of two conditions. Roughly one third of the sample was randomized into a condition to receive policy engagement opportunities via email (n=265), and two thirds were randomized to receive it via an online platform (n=584). The disproportionate randomization allowed us a greater sample size in the platform condition to test additional features. Enrollees received an average of 6.62 (SD = 6.48) opportunities to engage in January through September 2023. We tracked their contributions in response to those opportunities. We conducted a negative binomial regression to understand whether people responding via email and people responding via the platform contributed to policy engagement opportunities in different amounts.

**Results:**

Among the 746 researchers who had received at least one opportunity to engage, there was no difference in how many times researchers contributed to policy engagement opportunities by their email or platform condition, accounting for how many opportunities they were given, IRR = 0.92, SE = 0.19, z = -0.38, p = .71, 95%CI[0.61, 1.39]. Those receiving opportunities via email contributed an average of 0.37 times, and those receiving opportunities via the platform contributed an average of 0.34 times.

**Appendix 3: Gamification**

**Method:**

A sample of 694 researchers were randomized into one of four conditions: 1) receive opportunities via email; 2) receive opportunities via platform, with no additional features (community only); 3) receive opportunities via platform, with additional resources for engaging with policy (policy tools); and 4) receive opportunities via platform, with a badge/point system (gamification). During the time of these tests, researchers were randomized to one of these four conditions. After the data for this sub-test was closed for analysis, researchers were subsequently randomized into either the email or the platform conditions, as described in the previous Appendix. Therefore, the sample size for the analysis described in Appendix 2 is larger than this one. Data were captured and analyzed similarly to as described in Appendix 2.

**Results:**

Among the 639 researchers who had received at least one opportunity to engage, there were no significant differences in how many times researchers contributed to policy engagement opportunities by their condition, accounting for how many opportunities they were given. Notably, however, those who had been randomized to the Gamification condition contributed the least frequently of the four groups, as seen in the marginal means.

|  | IRR [LLCI, ULCI] | Marginal mean |
| --- | --- | --- |
| Email (ref) | - | 0.37 |
| Community only | 1.21 [0.71, 2.04] | 0.45 |
| Gamification | 0.78 [0.44. 1.37] | 0.29 |
| Policy tools | 1.00 [0.58, 1.73] | 0.37 |

**Appendix 4: Policy Scholars Award**

**Method:**

A sample of 76 identified researchers were awarded the RPC Scholars Award. The 76 researchers were determined to be the most active researchers in our virtual policy engagement platform. The 76 researchers were randomized into the extrinsic condition or control condition. Those in the extrinsic condition (*n* = 38) received personalized thank you letters acknowledging their contributions to the RPC, had access to a group CV, and were granted access to an exclusive platform community where they could engage with other active scholars (the other researchers in their condition). Data were captured and analyzed to test whether recipients in the extrinsic condition opened or clicked the email inviting them to connect with us on social media more than those in the control condition.

| Subjects | RPC: Connecting on social media |
| --- | --- |
| Bodies | Good morning, {{{Recipient.FirstName}}},  I hope your week is going well. We have heard from many network members they want to connect with one another and improve their networks with fellow policy engaged scholars. Thus, I wanted to draw attention to the use of our [Twitter](https://twitter.com/R2Policy) and [LinkedIn](https://www.linkedin.com/company/research-to-policy-collaboration/) followers list as a way to identify other network members online. In connecting with us, you will be visible to our partners as well. We strive to share info about related organizations and research in this space, an added benefit of connecting via social media.  We appreciate your efforts and its such an honor to work together to make an impact. |

**Results**

Researchers who were in the extrinsic test were 1.78 times more likely to open the email than researchers in the control group (OR = 1.78, SE = .26, *p* = .025, 95% CI [1.08, 2.97]) and opened the email an average of 1.78 times more (IRR = 1.78, SE = .15, *p* < .001, 95% CI [1.32, 2.40]). Additionally, researchers in the extrinsic test were 2.08 times more likely to click the link to RPC’s social media pages than those who weren’t in the test (OR = 2.08, SE = 26, *p* = .006, 95% CI [1.23, 3.48]), but did not click more times (IRR = 1.50, SE = .32, *p* = .205, 95% CI [0.82, 2.94]). These findings were largely to be expected because researchers in the test (who were subsequently randomized into the extrinsic reward or control conditions) were determined to be the most engaged 2.0 researchers in our network (threshold was 2 RR contributions with at least 1 since July 2022).

Within the extrinsic test (*n* = 76), those who were exposed to the extrinsic condition (i.e., the Higher Logic group) were not more likely to open the email (OR = 0.82, SE = .48, *p* = .682, 95% CI [0.32, 2.11]) nor did they open the email more times on average (IRR = 1.43, SE = .30, *p* = .231, 95% CI [0.80, 2.56]). Additionally, while there was a trend, being exposed to the extrinsic condition did not significantly predict higher odds of clicking on the social media link within the email (OR = 2.47, SE = .49, *p* = .067, 95% CI [0.95, 6.66]). Those in the extrinsic condition did not click the social media link more times, on average, than the control group (IRR = 2.02, SE = .43, *p* = .106, 95% CI [0.86, 4.77]). However, a large effect size was observed.

**Appendix 5: Intrinsic vs Extrinsic Motivation**

**Method:**

A sample of 781 warm contacts were invited to enroll in the RPC network via email, of which 625 were successfully delivered. Warm contacts are defined as researchers who had previously worked with us and those who indicated their interest working with us via a Google Form in 2021. The sample was randomized to receive one of the following messages. The subject line and email body were manipulated to emphasize motivations that were intrinsic or extrinsic in nature. Data were captured and analyzed to test whether recipients opened the email or whether recipients completed a consent form.

|  | Intrinsic motivations – 2019 network | Extrinsic motivations – 2019 network |
| --- | --- | --- |
| Subjects | **Make a difference** with the Research-to-Policy Collaboration | **Gain experience** with the Research-to-Policy Collaboration |
| Bodies | Hi {{recipient_first_name}},  You can gain **meaningful experiences** in the policy arena as part of the Research-to-Policy Collaboration Rapid Response Network. Researchers in our network have the opportunity **to give back to their community by sharing their research with decisionmakers who can act on it, establish meaningful relationships with collaborators who share their interests and values, and ultimately effect positive social change**.  Thank you for being part of our work during the 116th Congress! To remain in the network, please sign up again: {{contact_2021_baseline_survey_link_Mmag3U}}. | Hi {{recipient_first_name}},  You can gain **professional experiences** in the policy arena as part of the Research-to-Policy Collaboration Rapid Response Network. Researchers in our network have the opportunity **to expand their professional network, connect with new collaborators in relevant interest areas, build upon their CV with scholarly engagement, and increase their recognition with US policymakers.**  Thank you for being part of our work during the 116th Congress! To remain in the network, please sign up again: {{contact_2021_baseline_survey_link_Mmag3U}}. |

|  | Intrinsic motivations – Google form | Extrinsic motivations – Google form |
| --- | --- | --- |
| Subjects | **Make a difference** with the Research-to-Policy Collaboration | **Gain experience** with the Research-to-Policy Collaboration |
| Bodies | Hi {{recipient_first_name}},  You can gain **meaningful** experiences in the policy arena as part of the Research-to-Policy Collaboration Rapid Response Network. Researchers in our network have the opportunity to **give back to their community** **by sharing their research with decisionmakers who can act on it, establish meaningful relationships with collaborators who share their interests and values, and ultimately effect positive social change.**  Thank you for indicating your interest in joining the network. Please finish signing up here: {{contact_2021_baseline_survey_link_Mmag3U}}. | Hi {{recipient_first_name}},  You can gain **professional** experiences in the policy arena as part of the Research-to-Policy Collaboration Rapid Response Network. Researchers in our network have the opportunity to **expand their professional network, connect with new collaborators in relevant interest areas, build upon their CV with scholarly engagement, and increase their recognition with US policymakers.**  Thank you for indicating your interest in joining the network. Please finish signing up here: {{contact_2021_baseline_survey_link_Mmag3U}}. |

Result:

Data were analyzed using a logistic regression. There was no effect of intrinsic condition on whether the recipient opened the email (OR = 0.93, SE = .14, z = -0.48, p = .63, 95%CI[.69, 1.24]), clicked the links (OR = 0.93, SE = .18, z = -0.36, 95%CI[0.65, 1.35]), or completed the consent form (OR = 0.93, SE = .18, z = -0.36, 95%CI[0.65, 1.35]). Data were also analyzed using a negative binomial regression. There was no effect of extrinsic condition on whether the recipient opened the email (IRR = 1.21, SE = .17, z = 1.32, p = .19, 95%CI[0.91, 1.59]).

**Appendix 6:** **Recruitment 4 - time**

**Method:**

A sample of 1,435 researchers who were either enrolled in the 2019 network or who had indicated previous interest in a Google form were invited to enroll in the RPC network via email. The sample was randomized to receive one of the following messages. The subject line and email body were manipulated to emphasize low time commitment to participate because time commitment is one of the primary barriers to engaging in policy. We expected messages emphasizing low time commitment to outperform controls.

|  | Emphasizing low time commitment – 2019 network | Control – 2019 network |
| --- | --- | --- |
| Subjects | **Efficient** way to get research to policymakers | **Effective** way to get research to policymakers |
| Bodies | Hi {{recipient_first_name}},  Just following up on my invitation from last semester to join our [Rapid Response Network,](https://research2policy.org/) **which involves a minimal time commitment.**  [Our team](https://research2policy.org/who-we-are/) aims to create a pathway for policy engagement with **minimal burden on researchers’ time. We do all the legwork** to identify the right fit with congressional offices' needs, handle scheduling and administrative demands, **and consolidate network members’ insights into timely responses for offices.** When there are opportunities for meetings with staffers, **they tend to be brief (15-30 minutes)** and are facilitated by a policy associate.  It is **quick** and **simple** to re-enroll in the Network at the following link. The form will ask you to first consent to participating in a study that allows us to explore best practices for supporting researchers’ policy efforts. Then, we prompt for contact information and research interests so we can send you relevant opportunities, and finally a brief, voluntary evaluation survey. We ask that you please re-enroll so we are up-to-date on your research experience and contact information.  {{contact_2021_baseline_survey_link_Mmag3U}} | Hi {{recipient_first_name}},  Just following up on my invitation from last semester to join the [Research-to-Policy Collaboration](https://www.research2policy.org/) Rapid Response Network.  [Our team](https://www.research2policy.org/who-we-are) implements a model for bridging research and policy by **helping researchers communicate scientific evidence and build trusting relationships with policymakers.** Joining the network is an opportunity to help create resources for or meet with policy staff. We connect researchers with legislators based on a strong match with our researchers’ expertise and favorable political timing.  You can re-enroll the Network using the following link. The form will ask you to first consent to participating in a study that allows us to explore best practices for supporting researchers’ policy efforts. Then, we prompt for contact information and research interests so we can send you relevant opportunities, and finally a brief, voluntary evaluation survey. We ask that you please re-enroll so we are up-to-date on your research experience and contact information.  {{contact_2021_baseline_survey_link_Mmag3U}} |

|  | Emphasizing low time commitment – Google form | Control – Google form |
| --- | --- | --- |
| Subjects | **Efficient** way to get research to policymakers | **Effective** way to get research to policymakers |
| Bodies | Hi {{recipient_first_name}},  Just following up on your indicated interest to join our [Rapid Response Network,](https://research2policy.org/) **which involves a minimal time commitment.**  [Our team](https://research2policy.org/who-we-are/) **aims to create a pathway for policy engagement with minimal burden on researchers’ time.** We do all the legwork to identify the right fit with congressional offices' needs, handle scheduling and administrative demands, and consolidate network members’ insights into timely responses for offices. When there are opportunities for meetings with staffers, **they tend to be brief (15-30 minutes)** and are facilitated by a policy associate.  It is **quick** and **simple** to join the Network at the following link. The form will ask you to first consent to participating in a study that allows us to explore best practices for supporting researchers’ policy efforts. Then, we prompt for contact information and research interests so we can send you relevant opportunities, and finally a brief, voluntary evaluation survey.  {{contact_2021_baseline_survey_link_Mmag3U}} | Hi {{recipient_first_name}},  Just following up on your indicated interest to join the [Research-to-Policy Collaboration](https://www.research2policy.org/) Rapid Response Network.  [Our team](https://www.research2policy.org/who-we-are) **implements a model for bridging research and policy by helping researchers communicate scientific evidence and build trusting relationships with policymakers.** Joining the network is an opportunity to help create resources for or meet with policy staff. We connect researchers with legislators based on a strong match with our researchers’ expertise and favorable political timing.  You can join the Network using the following link. The form will ask you to first consent to participating in a study that allows us to explore best practices for supporting researchers’ policy efforts. Then, we prompt for contact information and research interests so we can send you relevant opportunities, and finally a brief, voluntary evaluation survey.  {{contact_2021_baseline_survey_link_Mmag3U}} |

**Results:**

Data were analyzed using logistic regression. There was no effect of time emphasis condition on whether the recipient opened the email (OR = 0.978, SE = 0.11, z = -0.19, p = .85, 95%CI[0.78, 1.23]), clicked the links (OR = 0.89, SE = 0.25, z = -0.43, p = .67, 95%CI[0.51, 1.54]), entered the consent form (OR = 0.81, SE = 0.31, z = -0.57, p = .57, 95%CI[0.38, 1.69]), or completed the consent form (OR = 0.94, SE = 0.33, z = -0.18, p = .86, 95%CI[0.47, 1.88]). Data were also analyzed using negative binomial regression. There was no effect on how many times the email was opened (IRR = 0.87, SE = 0.10, z = -1.22, p = .22, 95%CI[0.69, 1.09]) or the links clicked (IRR = 0.72, SE = 0.24, z = -0.96, p = .34, 95%CI[0.37, 1.40]).

**Appendix 7: RR trial 4 - urgency**

**Method:**

A sample of 184 Researchers who contributed to at least 1 rapid response during the RR4 series were asked to respond to another rapid response. The subject line and body of the email were manipulated to emphasize the urgency of the request. The sample was randomized to receive one of the following messages. We expected the emails emphasizing the urgency of the request to outperform controls.

|  | Emphasizing the urgency of the request | Control |
| --- | --- | --- |
| Subjects | RPC: **Urgent -** [] resource request | RPC: [] resource request |
| Bodies | Hello,  We met with a congressional office interested in []. We are reaching out to see if you or other members can **quickly** provide insights on the following by [] **at the latest**. **They need this information soon because [REASON].**  [] | Hello,  Hope your week is going well! We recently met with a congressional office interested in []. We are reaching out to relevant members of the Rapid Response Network to see if you or other members can provide any insights from the research literature on the following questions by [].  [] |

**Result:**

There was no impact of marking something as urgent on whether someone contributed to policy engagement opportunities, OR = 0.71, RSE = 0.76, z = -0.32, p = .75, 95%CI[0.09, 5.7].

**Appendix 8: Emphasizing benefits backfired**

**Method:** A sample of 164 people were emailed a total of 287 times, 29 of whom (accounting for 55 emails) contributed during this time. The subsample of those who contributed were retained for analysis. These people were emailed a request for research using one of two adapted scripts (see table). Data were captured and analyzed to test whether recipients opened the email or contributed to the rapid response.

|  | Explain the benefits | Control includes niceties and modest expectations |
| --- | --- | --- |
| Subject | RPC, []: Professional opportunity | RPC: RPC, []: Engagement opportunity |
| Body | Hello!  Researchers in our network have reported that **they benefit from learning** how to develop policy-relevant, high-impact research questions and achieving social impact with their research. We recently met with congressional offices interested in []. We’re reaching out to you because of your relevant interest/experience in this topic, which may provide you an opportunity to work on these skills.  Specifically, the offices are interested in learning more about the following:  It is perfectly fine if you do not have all the answers! We are interested in any input and/or resources that you’re able to offer related to the questions above—**this is a great opportunity to practice science communication**. Timelines in Congress are constantly in flux, so please email us your reply by []. | Hello!  **Hope your week is going well,** and if you are on a semester system that the new year is off to a good start, whether online or in person! We recently met with congressional offices interested in []. We’re reaching out to you because of your relevant interest/experience in this topic.  Specifically, the offices are interested in learning more about the following:  It is perfectly fine if you do not have all the answers! We are interested in any input and/or resources that you’re able to offer related to the questions above—**anything you can provide helps.** Timelines in Congress are constantly in flux, so please email us your reply by []. |

**Results:** There was no effect of the benefit framing on whether someone opened the email, OR = 1.21, RSE = 1.41, z = 0.16, p = .87. But there was an effect on how many times it was opened, such that those who got the email emphasizing the benefit opened the email 73% more times than those who received the control email, IRR = 1.73, RSE = 0.47, z = 2.01, p = .045, 95%CI[1.01, 2.96]. Those who received the control email were 4x as likely to contribute than those who received the benefit email, OR = 4.08, RSE = 2.55, z = 2.25, p = .024, 95%CI[1.20, 13.9].

**Appendix 9: Recruitment - meeting offer**

**Method:**

A sample of 879 cold contacts were invited to enroll in the RPC network via email. Cold contacts are defined as researchers the RPC identified through a search of university and research center websites who may be interested in joining the Rapid Response Network. Cold contacts were randomized to receive an offer to meet with us if they had any questions. If they were in the “meeting offered” condition, their email included an invitation to join meet & greet hours including times and a zoom link. Those in the control condition did not receive that offer. Data were captured and analyzed to test whether recipients opened the email or completed a consent form.

|  | Offering a meeting | No meeting offered - control |
| --- | --- | --- |
| Subjects | Invitation to the Research-to-Policy Collaboration Network | Invitation to the Research-to-Policy Collaboration Network |
| Bodies | Hello {{recipient_first_name}} {{recipient_last_name}},  We are excited to invite you to join the Research-to-Policy Collaboration Rapid Response Network! The Research-to-Policy Collaboration (RPC) is a model for bridging research and policy that emphasizes partnerships between legislative staff and researchers in the RPC Rapid Response Network.  **If you have any questions, you may join any of our meet & greet hours throughout August (times and zoom details are beneath the signature line). If these times don’t work, just let us know and we can schedule another time.**  To join the RPC Rapid Response Network, please complete your registration here: {{contact_2021_baseline_survey_link_Mmag3U}}. | Hello {{recipient_first_name}} {{recipient_last_name}},  We are excited to invite you to join the Research-to-Policy Collaboration Rapid Response Network! The Research-to-Policy Collaboration (RPC) is a model for bridging research and policy that emphasizes partnerships between legislative staff and researchers in the RPC Rapid Response Network.  To join the RPC Rapid Response Network, please complete your registration here: {{contact_2021_baseline_survey_link_Mmag3U}}. |

**Results:**

Data were analyzed using a logistic regression. There was no effect of offering a meeting on whether the recipient opened the email (OR = 1.05 SE = .14, z = 0.39, p = .70, 95%CI[.81, 1.38]) or completed the consent form (OR = 0.94, SE = .50, z = -0.12, p = .91, 95%CI[.33, 2.70]). Data were also analyzed using a negative binomial regression. There was no effect of not offering a meeting on whether the recipient opened the email (IRR = 0.98, SE = .13, z = -0.12, p = .91).

**Appendix 10: Personalizing**

**Method:**

521 emails were sent to 288 research network members who contributed to at least 1 rapid response during the RR2 timeframe asking them to contribute to another rapid response. The subject line and body of the email were personalized to the recipient in the subject line and body. The sample was randomized to receive one of the following messages. Data were captured and analyzed to test whether recipients opened the email or contributed to a resource request. We expected personalized emails to outperform controls.

|  | Personalized email | Control |
| --- | --- | --- |
| Subjects | **{{recipient_first_name}} {{recipient_last_name}}**: [] resource request | [] resource request |
| Bodies | Hello **{{recipient_first_name}}**,  Hope your week is going well! We recently met with congressional offices interested in []. **We have identified you from our Rapid Response Network as being among those who have experience related to this area, and are interested in any insights you can provide** from research about the following topics.  Specifically, the offices want to learn more about the following: [] | Hello,  Hope your week is going well! We recently met with congressional offices interested in [] **We are reaching out to the members of the Research-to-Policy Collaboration’s Rapid Response Network to see if anyone can provide any insights** from the research literature on the following topics.  Specifically, the offices want to learn more about the following: [] |

**Results:**

There was no effect of personalization on whether someone opened that email at all (IRR = 1.42, RSE = 0.88, z = 0.57, p = .57), how many times they opened (IRR = 1.35, RSE = 0.28, z = 1.43, p = .15, 95%CI[0.90, 2.02]), or whether they contributed to that response (IRR = 1.21, RSE = 0.45, z = 0.52, p = .61, 95%CI[0.58, 2.52]). Personalization was indeed higher in each of these than the control, however, these results trend in the same direction with the full sample.

**Appendix 11: Recruitment - personal narrative**

**Method:**

A sample of 1,689 researchers were invited to enroll in the RPC network via email, of which 1,363 were successfully delivered. Researchers were randomized to receive an email that either emphasized low time commitment or offered a personal narrative relative to the work. The subject line was manipulated to emphasize the low time commitment to participate because time commitment is one of the primary barriers to engaging in policy. The body was manipulated to detail a narrative regarding why the co-founder of the RPC finds this work to be important. We expected the low time commitment emphasis and the narrative to outperform the controls.

|  | Email offering a personal narrative | Email emphasizing low time commitment |
| --- | --- | --- |
| Subjects | **Opportunity to engage** in and impact policy | **Minimal time commitment** for policy impact |
| Bodies | Hi {{recipient_first_name}},  I wanted to reach out because I'd like your involvement in communicating research to policymakers. **Personally, I became a researcher because I wanted to make a difference in the lives of real people.** **But I've often felt disappointment in realizing that research doesn't always get into the hands of people who can use it for social change.** The gap between research and policy has driven my career in creating a feasible and time-efficient pathway for researchers to communicate with policymakers. Hence, the Research-to-Policy Collaboration provides a way for researchers to respond to policymakers' requests for evidence and occasionally meet when there's a great match between their areas of interest. This responsive, apolitical approach to research translation is critical to our success, and **it's rewarding to see research achieve its intended social impact.** I hope you'll consider joining us in this mission. | Hi {{recipient_first_name}},  I’m Taylor Scott, co-director of the Research-to-Policy Collaboration. The RPC is a theory-driven, replicable, and non-partisan approach that aims to increase policymakers’ use of research evidence by building trusting relationships between policy and science communities. We aim to provide non-partisan support to legislative staff by connecting them with relevant research-oriented scholars. Our goal is to carve a pathway for scholars to engage with policy so research can move towards its intended impact. To achieve that, we have created a feasible and time-efficient pathway for researchers to communicate with policymakers. Hence, the Research-to-Policy Collaboration provides a way for researchers to respond to policymakers' requests for evidence and occasionally meet when there's a great match between their areas of interest. This responsive, apolitical approach to research translation is critical to our success and impact. |

**Results:**

Controlling for the other conditions, there was a significant effect of narrative, but not of time. The odds of opening the email changed by a factor of 1.33 among those who received the narrative email body relative to those who received the standard email body, OR = 1.33, SE = 0.14, z = 2.61, p = .009, 95%CI[1.07, 1.64]. There was no effect of the time subject line, OR = 1.09, SE = 0.12, z = 0.70, p = .49, 95%CI[0.87, 1.34].

**Appendix 12: PA intro trial**

**Method:**

A sample of 470 researchers who were enrolled and valid in the Rapid Response Network were invited to click a link to learn more about new RPC policy associates. The sample was randomized to receive one of the following messages. The subject line was manipulated to emphasize the background of the policy associates. The body was manipulated to detail a narrative regarding why the policy associates find this work to be important. Data were captured and analyzed to test whether recipients opened or clicked the email.

|  | Email emphasizing narrative | Email emphasizing credibility |
| --- | --- | --- |
| Subjects | The **backstories** of our new Policy Associates | The **experience** our new Policy Associates bring |
| Bodies | Happy Tuesday, RPC Rapid Response Network!  We are excited to welcome TWO new policy associates to our team, Antoine Lovell and Mallory Matheson, **both of whom are invested in improving the impact of research.** With their help, we can coordinate more requests from Congress, so please watch for emails from them with new opportunities.  **Antoine Lovell** **is passionate about RPC’s mission because his work in intervention research has demonstrated how evidence can be used as a tool to mitigate and alleviate poverty for society’s most vulnerable.**  **Mallory Matheson has been drawn to this work since 2017, when she was excited to see policy recommendations made by her research team actually used by policymakers, and she learned how rare that is.**  You can read more about their stories here: <https://www.research2policy.org/who-we-are> | Happy Tuesday, RPC Rapid Response Network!  We are excited to welcome TWO new policy associates to our team, Antoine Lovell and Mallory Matheson, **both of whom are highly qualified to work in the research-policy sector.** With their help, we can coordinate more requests from Congress, so please watch for emails from them with new opportunities.  **Antoine Lovell** is a doctoral candidate (for three more weeks, when he is expected to defend his dissertation!) at Fordham University Graduate School of Social Service with extensive experience working with legislators and the New York Housing Authority.  **Mallory Matheson** **has experience with nearly every step in the political process both within and outside of the government, including campaigns, policy advising and lobbying, and evaluating programs.**  You can read more about their qualifications here: <https://www.research2policy.org/who-we-are> |

**Result**

There was no effect of the condition on whether the recipient opened at all (OR = 0.90, SE = 0.17, z = -0.55, p = .58, 95%CI[0.63, 1.30]), nor how many times they opened (IRR = 1.08, SE = 0.14, z = 0.61, p = .54, 95%CI[0.84, 1.39]). There was not an effect of the condition on whether they clicked at all (OR = 0.54, SE = 0.17, z = -1.88, p = .06, 95%CI[0.28, 1.03]), but there was a (not robust) effect on how many times they clicked, such that the Qualifications email links received about half as many clicks as the Narrative email links (IRR = 0.47, SE = 0.18, z = -1.99, p = .047, 95%CI[0.23, 0.99]).

**Appendix 13: Platform launch email**

**Method:**

A sample of 446 researchers were invited to join a new online platform associated with the network of which they were part. They had to take action to finish their enrollment. They were randomized evenly to receive an email that enthusiastically introduced the platform and emphasized its cutting-edge nature or an email that used "authentic” cues in introducing the platform and acknowledged the efforts required to participate. For example, the enthusiastic email does not acknowledge that this platform will disrupt their existing workflows, and the less enthusiastic email acknowledges that the adjustment will take time. Data were captured and analyzed to test whether recipients opened or clicked the email and whether recipients joined the platform.

| Authentic, emphasized barriers | Enthusiastic |
| --- | --- |
| {{recipient_first_name}}  We are pleased to invite you to join the Research-to-Policy Collaboration’s exciting new platform for the Rapid Response Network! This platform will replace our current method of emailing you with research requests**.** **We know that this new process will take some adjusting to (for all of us!), but we’ve tried to make it as easy as possible.** We’re hoping that by switching to a community-based platform instead of individual emails, network members will be able to meet each other and collaborate more directly on research requests.  [content about the platform, consistent across conditions]  Thank you for your continued support of RPC’s mission and work to advance evidence-based policy—we couldn’t do this without you. **We recognize it will take some time to adjust, and it might be a bit confusing.**  All my best,  [sign-off] | {{recipient_first_name}}  We are **excited to invite you to join as a founding contributor** in the Research-to-Policy Collaboration’s new rapid response platform. **We believe this is going to revolutionize the way researchers can engage in policy efforts across the country.** This platform will replace our current method of emailing you with research requests**.** By switching to a community-based platform instead of individual emails, you and your fellow researchers will be able to meet each other and collaborate more directly on research requests.  [content about the platform, consistent across conditions]  Thank you for your continued support of RPC’s mission and work to advance evidence-based policy—we couldn’t do this without you.  [sign-off] |

**Results:**

There was a significant effect of the email on whether recipients opened the email (OR = 0.57, SE = 0.11, z =-2.77, p =.006, 95%CI[0.38, .85]) and on how many times they opened the email (IRR = .68, SE = 0.08, z = -3.25, p = .001, 95%CI[0.53, .86]). Authenticity (compared to enthusiastic condition) decreased probability of opening the email, and the number of opens, by about half. There was no effect of authenticity cues on whether a recipient clicked a link in the email (OR = 0.77, SE = 0.16, z = -1.27, p = .20, 95%CI[0.51, 1.15]), nor how many times they clicked (IRR = 0.86, SE = 0.18, z = -0.71, p = .48, 95%CI[0.58, 1.28]). Additionally, there was a significant effect of authenticity on likelihood to join the platform. Specifically, authenticity decreased likelihood to join the platform by about half (OR = 0.65, SE = 0.13, z =-2.15, p =.032, 95%CI[0.43, .96]), but no effect of authenticity on when recipients joined the platform (B=.200, SE = 0.32, t =.63, p =.52, 95%CI[-0.42, .83]).

**Appendix 14: RR trial 3 - empathy**

**Method:**

A sample of 247 people were emailed a total of 585 times, 29 of whom (accounting for 85 emails) contributed during this time. The subsample of those who contributed were retained for analysis. These people were emailed a request for research using one of two adapted scripts (see table). We expected the emails empathizing with time constraints to outperform controls.

|  | Empathizing with time constraints | Control: Highlight expertise match |
| --- | --- | --- |
| Subject | RPC: Resource request | RPC: Help research impact policy |
| Body | Hello,  There is a new opportunity to translate research for policymakers and **we’re hoping it’s manageable with your other commitments**. We recently met with a congressional office interested in []. **We are reaching out to members** of the Research-to-Policy Collaboration’s Rapid Response Network to see if you or other members can provide any insights from the research literature on the following by [].  We are **interested in any input** or resources you can offer related to the questions above. If you know someone who might want to provide input, please send us their email address.  **It can be tough to balance** all the distinct hats you probably wear, and we **appreciate** you being part of the Rapid Response Network. Thank you in advance for your time and engagement!  Best regards, | Hello,  We have another exciting request from a legislative office that **we believe is a good fit for you** to draw from your research experience to achieve social impact. We recently met with a congressional office interested in []. **Given your relevant experience,** we are interested in any research insights you can provide about the following topics by [].  We’d be **delighted** if there are resources you have already gathered for your own work that you would like to share. If you know someone who might want to provide input, please send us their email address.  **We are here to support you** in this process, and **hope it is a good match** with your expertise. Thanks in advance for your time and engagement!  Warmly, |

**Results:**

People who received the empathetic email were 2.3x as likely to contribute than those who received the expertise email, OR = 2.36, RSE = 0.98, z = 2.06, p = .039, 95%CI[1.04, 5.32]. Importantly, during this trial and beyond, we received notes from participants acknowledging the request was outside of their knowledge area and requesting more appropriate targeting methods. Perhaps this perceived mismatch counteracted any feelings of importance they would have felt had it been a better match. In contrast, the message that empathized with their time constraints and acknowledged the value of their time would not have been negatively affected in the same way by a mismatch.

**Appendix 15: Launch event RSVP**

**Method:**

A sample of 432 researchers were invited to a networking event via email. They were randomized to receive an email that either acknowledged barriers to engagement or channeled enthusiasm. Their opening of the email, clicking the link to RSVP, and completion of the RSVP form were tracked. Data were tracked for one week.

|  | Acknowledged barriers (“authentic”) | Enthusiasm |
| --- | --- | --- |
| Subject | Invitation: RPC Speed Networking Launch Event | Invitation: RPC Speed Networking Launch Event! |
| Body | Hey {{recipient_first_name}},  I am reaching out to invite you to our virtual platform launch party and speed networking session on Thursday, July 14th, from 1:00-2:00pm ET. Register [here](https://psu.zoom.us/meeting/register/tJYqfuChrjssGNTzz3NGLHRu5yWN5t9Mdkpa)!  As you may know, **my team at RPC and I** recently launched a cutting-edge platform for the Rapid Response Network that **we are hoping** will replace our current method of emailing you with requests. **Because of my role at RPC, I've seen how the email process works and honestly, it can be a bit tough to follow.** With this platform, we're hoping to **revise that process and make it easier** for you to engage and **feel more connected** with other scholars interested in policy. It's been a long time in the works but I'm feeling **pretty proud** of the platform and think it can do a lot to help form **relationships** and really **revolutionize** the way scholars engage with policy across the country.  This launch event **is one of many ways the platform will help you** connect directly with other policy-engaged scholars. Through a few rotating breakout room sessions, you will get to meet fellow Rapid Response Network members who have similar topic area expertise (e.g., Criminal Justice, Economics, etc.).  **You can** register here: link  **I would love it if you could join us and help make our launch a success!** | Hello {{recipient_first_name}},  We are pleased to invite you to a virtual platform launch party and speed networking session on **T**hursday, July 14th, from 1:00-2:00pm ET. Register [here](https://psu.zoom.us/meeting/register/tJYqfuChrjssGNTzz3NGLHRu5yWN5t9Mdkpa)!  As you may know, **we** recently launched a cutting-edge platform for the Rapid Response Network that will replace our current method of emailing you with requests. **This platform provides a place for you to connect with and learn from other Network members** – you can add others as contacts, send private messages, interact in **topic-based communities**, and **collaborate** directly on requests for research. We believe this has the power to **revolutionize** the way researchers can engage in policy efforts across the country, and we are **excited** for you to be one of the founding members.  This launch event is yet another opportunity to connect directly with other policy-engaged scholars. Participants will be able to join a few rotating Zoom breakout room sessions based on topic area expertise (e.g., Criminal Justice, Economics, etc.) to meet fellow Rapid Response Network members, **share thoughts on the field,** and explore **why** everyone joined RPC and how their **respective experiences** inform their approach to bridging the research-policy gap.  **Please** register for the event here: link  **Looking forward to seeing you there!** |

**Results:**

There was no effect of authenticity on whether a recipient opened an email (OR = 0.85, SE = 0.16, z =-0.87, p = .39, 95%CI[0.58, 1.23]), nor how many times they opened (IRR = 1.05, SE = 0.15, z = 0.37, p = .71, 95%CI[0.80, 1.39]). There was also no effect of authenticity on whether a recipient clicked a link in the email (OR = 0.57, SE = 0.19, z = -1.64, p = .10, 95%CI[0.29, 1.11]) or how many times they clicked (IRR = 0.74, SE = 0.28, z = -0.81, p = .42, 95%CI[0.35, 1.54]). Note, however, that all of these are pointing in the direction that authenticity is underperforming compared to the enthusiastic email. That finally comes to fruition in the RSVPs, in which the odds of a recipient of **the authentic email registering for the event was half the odds for a recipient of the enthusiastic email registering for the event,** OR = 0.47, SE = 0.18, z = -1.96, p = .05, 95%CI[0.22, 1.00]. Five percent of the authentic recipients registered, as compared to 10% of the control recipients.

**Appendix 16: Recruitments - warm hand-off**

**Method:**

In a series of four tests, researchers were emailed an invitation to join the Rapid Response Network. In the first test, recipients were randomized to receive the standard recruitment email from an internal team member or to receive a slight adaption in which we mentioned they were referred to us by someone in their field. In subsequent tests, they were randomized into equal groups to receive an email that appeared to be sent by someone internal to the team (standard) or someone in their field (referral). Data were captured and analyzed to test whether recipients opened or clicked the email and whether recipients entered or completed a consent form.

|  | Control | Referral |
| --- | --- | --- |
| Education | Sender: Internal  Subject line: Invitation to a policy rapid response network  Hi {{recipient_first_name}},  We’re working **to improve** the impact of research related to educational equity and **think your work** could meaningfully contribute to policy impact. I’m Brittany Gay, the Associate Director of Implementation Science and want to invite you to the Research-to-Policy Collaboration (RPC) **Rapid Response Network**. | Sender: Internal  Subject line: **Recommended by {{referee}}:** Invitation to a policy rapid response network  Hi {{recipient_first_name}},  We’re working **with {{referee}}** on improving the impact of research related to educational equity. **{{referee} recommended** that we reach out to you because your work could meaningfully contribute to policy impact. I’m Brittany Gay, the Associate Director of Implementation Science of the Research-to-Policy Collaboration (RPC). |
| Education - again | Sender: **Internal**  Hi {{recipient_first_name}},  Just wanted to follow up on **my invitation from** a few weeks ago to the Research-to-Policy Collaboration Rapid Response Network! The Research-to-Policy Collaboration (RPC) is a model for bridging research and policy. That is, we help create and sustain partnerships between legislative staff and researchers, and call on scholars in the RPC Rapid Response Network to provide empirical evidence that we highlight in policy briefs. **We are currently working** on educational equity projects in particular, **and we** think you would be a great fit for the Network given your relevant work. | Sender: **In their field**  Hi {{recipient_first_name}},  Just following up on **an invitation you received** a few weeks ago to the Research-to-Policy Collaboration Rapid Response Network! The Research-to-Policy Collaboration (RPC) is a model for bridging research and policy. **Their team** helps create and sustain partnerships between legislative staff and researchers, and calls on scholars in the RPC Rapid Response Network to provide empirical evidence that we highlight in policy briefs. **I’m working with them** on educational equity projects in particular, and **{{referee}}** think you would be a great fit for the Network given your relevant work. |
| Climate | Sender: **Internal**  Hi {{recipient_first_name}},  I’d like to follow up on an invitation to join the Research-to-Policy Collaboration Rapid Response Network! The Research-to-Policy Collaboration (RPC) is a model for bridging research and policy. That is, we help create and sustain partnerships between legislative staff and researchers, and call on scholars in the RPC Rapid Response Network to provide empirical evidence that we highlight in policy briefs. **We are currently working** on environment/energy projects in particular, **and we** think you would be a great fit for the Network given your relevant work. | Sender: **In their field**  Hi {{recipient_first_name}},  I’d like to follow up on an invitation for you to join the Research-to-Policy Collaboration Rapid Response Network! The Research-to-Policy Collaboration (RPC) is a model for bridging research and policy. **Their team** helps create and sustain partnerships between legislative staff and researchers, and calls on scholars in the RPC Rapid Response Network to provide empirical evidence that we highlight in policy briefs. **Along with [two collaborators, in climate field], I am working with the RPC** on building out their portfolio on environment/energy projects in particular with seed funding from the PSU Institutes for Energy and the Environment. **We** think you would be a great fit for the Network given your relevant work. |
| Entrepreneurship | Sender: **Internal**  Hi {{recipient_first_name}},  I was hoping you would be interested in joining the Research-to-Policy Collaboration Rapid Response Network? The Research-to-Policy Collaboration (RPC) is a model for bridging research and policy. That is, we help create and sustain partnerships between legislative staff and researchers, and call on scholars in the RPC Rapid Response Network to provide empirical evidence and meet with offices. **We are currently** building out our work on entrepreneurship in particular, **and we** think you would be a great fit for the Network given your relevant work. | Sender: **In their field**  Hi {{recipient_first_name}},  I was hoping you would be interested in joining the Research-to-Policy Collaboration Rapid Response Network? The Research-to-Policy Collaboration (RPC), developed at Penn State University, is a model for bridging research and policy. **Their team** helps create and sustain partnerships between legislative staff and researchers, and calls on scholars in the RPC Rapid Response Network to provide empirical evidence and meet with offices. **They are currently** building out their work on entrepreneurship in particular, and I think you would be a great fit for the Network given your relevant work. |

**Results:**

Test 1: Name drop (N=129)

There was no effect of referral condition on whether the recipient opened the email (OR = 1.10, SE = 0.40, z = 0.25, p = .80, 95%CI[0.54, 2.23]), clicked the links (OR = 1.66, SE = 0.86, z = 0.97, p = .33, 95%CI[0.60, 4.59]), entered the consent form (OR = 1.96, SE = 1.07, z = 1.25, p = .21, 95%CI[0.68, 5.69]), or completed the consent form (OR = 1.36, SE = 0.78, z = 0.53, p = .59, 95%CI[0.44, 4.16]). There was also no effect on how many times the email was opened (IRR = 1.40, SE = 0.38, z = 1.23, p = .22, 95%CI[0.82, 2.40]) or the links clicked (IRR = 1.51, SE = 0.89, z = 0.70, p = .48, 95%CI[0.48, 4.79]).

Test 2: Direct referral; education topic (N=116)

There was no effect of the sender on whether they opened (OR = 1.08, SE = 0.42, z = 0.20, p = .84, 95%CI[0.50, 2.33]), or how many times they opened (IRR = 1.55, SE = 0.41, z = 1.65, p = 0.10, 95%CI[0.92, 2.61]). There was also no effect on whether the recipient clicked (OR = 2.21, SE = 1.29, z = 1.36, p = .17, 95%CI[0.70, 6.94]) or how many times they clicked (IRR = 1.90, SE = 1.19, z = 1.02, p = .31, 95%CI[0.56, 6.50]).

There was, however, a significant effect on enrollments, such that people who received the email from someone in their field were 9.12x as likely to complete the intake than those who received the email from an internal team member that name-drops the person in their field, OR = 9.12, SE = 9.83, z = 2.05, p = 0.40, 95%CI[1.10, 75.47].

Test 3: Direct referral; climate topic (N=94)

Despite similar open rates (OR = 0.91, SE = 0.40, z = -0.22, p = .82, 95%CI[0.38, 2.17]), relative to recipients who heard from the internal team member, those who heard from someone in their field were (practically/clinically, but not statistically significant) nearly 3x as likely to open the consent form (OR = 2.88, SE = 1.67, z = 1.83, p = .068, 95%CI[0.93, 8.97]), and 3.5 times as likely to complete the intake (OR = 3.47, SE = 2.44, z = 1.77, p = .076, 95%CI[0.88, 12.76]).

Test 4: Direct referral; entrepreneurship topic (N=202)

There was no effect of sender on whether the email was opened (OR = 1.20, SE = 0.34, z = 0.63, p = .53, 95%CI[0.69, 2.08), how many times it was opened (IRR=1.60, SE=0.43, z=1.75, p=.08, 95%CI[0.94, 2.72]), whether it was clicked (OR=2.11, SE = 0.88, z=1.78, p=.075, 95%CI[0.93, 4.80]), or whether someone enrolled (OR=1.80, SE=1.03, z=1.02, p=.31, 95%CI[0.58, 5.55]). However, there was a significant effect on how many times it was clicked (IRR=3.05, SE=1.52, z=2.23, p=.025, 95%CI[1.15, 8.12]), such that recipients of emails sent from someone in their field were clicked more frequently than those sent from an internal team member.

**Appendix 17: Gamification nudge**

**Method:**

A sample of 151 researchers were sent an email reminding them about a feature in the platform that allows them to earn points and badges for participating. Two characteristics were manipulated in this test. The first characteristic is the medium: recipients either received a link to a video about the feature, or a text paragraph. The second characteristic that was manipulated was the language frame: recipients either received language that pulled on “leadership communication” principles, or did not (the control). The script of the video mirrored that of the text in the “text” condition. We expected leadership communication to convey appropriate recognition. Data were captured and analyzed to test whether recipients clicked a link embedded in the email.

| Control | Subject line:  RPC: Tutorial of Badge System  Body:  Hello {{recipient_first_name}},  We’d like to tell you about the platform’s points and badge feature. This feature is meant to provide a way for network members to communicate their commitment to policy engagement.  On [your own](https://rapidresponse.research2policy.org/profile/contributions/contributions-achievements) or [others’ (e.g., my)](https://rapidresponse.research2policy.org/profile/contributions/contributions-achievements?UserKey=be3e70bc-c96c-4687-b908-3dbf161284c1) profiles, hover your cursor over a badge to see how it was earned, or click on it to see all available badges. [This page](https://rapidresponse.research2policy.org/achievements) lists the available badges and how to earn them. You will be alerted via email when you have received a new badge on your profile.  Some badges are given for completing your profile or adding other network members as contacts; other badges are based on how many times you have responded to Rapid Response requests since the platform launched, and how much you engage with the platform overall.  Thank you, I hope this was helpful. We welcome suggestions.  All my best,  Jessica |
| --- | --- |
| Leadership communication | Subject line:  RPC Platform: Feature to Recognize Your Contributions  Body:  Hello {{recipient_first_name}},  Corresponding with best practice for online forums, we are delighted to showcase the points and badge features on the new platform. We created this system so that our colleagues leading in policy efforts are recognized for their inspiring commitment to social impact.  On [your own](https://rapidresponse.research2policy.org/profile/contributions/contributions-achievements) or [others’ (e.g., my)](https://rapidresponse.research2policy.org/profile/contributions/contributions-achievements?UserKey=be3e70bc-c96c-4687-b908-3dbf161284c1) profiles, hover your cursor over a badge to see how it was earned, or click on it to see all available badges. [This page](https://rapidresponse.research2policy.org/achievements) outlines all the available badges and what it means someone has achieved if you see it on their profile. When you earn a badge, you will receive an email alert and then see the badge on your profile, too.  Some of these badges are awarded for making the platform a better experience for everyone, like finishing your profile and adding other network members as contacts. Other badges are awarded for your meaningful policy engagement, like how many times you have responded to Rapid Response requests since the platform launched, and how much you engage with the platform overall. These serve as a testament to supporting policymakers and other scholars in this space.  This is just the first version of the badge system, and we welcome your suggestions for future renditions.  Thanks so much, we look forward to making an impact alongside you.  All my best,  Jessica |

**Results:**

There was no difference in number of clicks based on the medium (IRR = 0.76, SE = 0.58, z = -0.36, p = .72, 95%CI[0.17, 3.42]), but those who received the Leadership Communication language clicked the links 6x as many times as those who received the Control language *but note the high standard error* (IRR = 6.05, SE =5.42, z = 2.01, p = .044, 95%CI[1.04, 35.02]).

**Appendix 18:** Recruitment 3 - credibility types

**Method:**

A sample of 1643 researchers were emailed with an invitation to participate in a research-policy network. They were randomized to receive one of three messages. These messages emphasized three different aspects of credibility to understand if those were important in encouraging scholars to enroll. The “academic credibility” message emphasized the team’s track record publishing papers in peer-reviewed journals and receiving research grants, the “applied credibility” message emphasized the team’s reach within Congress, and the “social credibility” message emphasized the team’s reach with other scholars. Data were captured and analyzed to test whether recipients opened or clicked the email and whether recipients enrolled in the network.

| Academic | I want to follow up on my invitation to join a Rapid Response Network to advance the translation of scientific knowledge—apologies for the nudge. In addition to connecting policymakers with researchers, the Research-to-Policy Collaboration (RPC) also conducts rigorous research to investigate how researcher-policymaker relationships can support evidence-based policy. This research has been published in [American Psychologist](https://doi.apa.org/doi/10.1037/amp0000466), [Proceedings of the National Academies of Sciences](https://doi.org/10.1073/pnas.2012955118), and [Evidence & Policy](https://doi.org/10.1332/174426421X16134931606126). Generous support for our work comes from the W.T. Grant Foundation and NSF.  Researchers in the Rapid Response Network get to share their scholarship with policymakers while simultaneously contributing to novel research on how we can accelerate the uptake of scholarly work.  [info about and link to enroll] |
| --- | --- |
| Applied | I want to follow up on my invitation to join a Rapid Response Network for achieving social impact of research in public policy—apologies for the nudge. During the last Congress (2019-2020), we made a [measurable impact](http://wtgrantfoundation.org/congressional-use-of-evidence-can-be-improved-reflections-from-a-trial-of-the-research-to-policy-collaboration-model) on policymakers’ value and use of research by holding over 450 meetings with over 200 legislative offices. We responded to the pandemic by [distributing](https://magazine.hhd.psu.edu/2020/11/30/mincing-words-making-research-available-to-policymakers-during-covid-19/) over 75 relevant [science syntheses](https://www.research2policy.org/issues) to several thousand state and federal legislative offices, prompting even more researcher-policymaker connections.  As we expand to achieve impact in more policy areas, we need researchers in diverse disciplines to contribute to a rapid response network so that we can respond to a wide range of policymakers’ questions that inform their social policy decisions.  [info about and link to enroll] |
| Social | I want to follow up on my invitation to join your policy-oriented colleagues in our Network—apologies for the nudge. Over 500 researchers across the nation, and even beyond US borders, have joined. Our Rapid Response Network members represent nearly every state in the country and are already responding to policymakers’ requests for research in a wide variety of areas, such as [education](https://www.research2policy.org/covid-19), [victimization](https://www.research2policy.org/violence), and [criminal justice](https://www.research2policy.org/police-reform).  By joining our growing Rapid Response Network, you will also have the opportunity to work with colleagues from across the nation, at your own pace, to translate current research for policymakers.  [info about and link to enroll] |

**Results:**

There was no effect of Credibility on whether the email was opened (χ^2^ (2) = 3.75, p = .15) or how many times it was opened (χ^2^ (2) = 3.46, p = .18). There was, however, an effect of credibility on whether internal links were clicked (χ^2^ (2) = 8.18, p = .017), and how many times it was clicked (χ^2^(2) = 8.14, p = .017). The policy email had the most clicks. Compared to the social email, the policy email was 3.09x as likely to be clicked (OR = 3.09, SE = 1.36, z = 2.56, p = .01, 95%CI[1.30, 7.33]) and clicked 5.27 times more frequently (IRR = 5.27, SE = 3.13, z = 2.80, p = .005, 95%CI[1.64, 16.88]). Compared to the academic email, the policy email was 2.14x as likely to be clicked (OR = 2.14, SE = 0.83, z = 1.96, p = .05, 95%CI[1.00, 4.59]), but no more or less frequently so (IRR = 2.71, SE = 1.54, z = 1.75, p = .08, 95%CI[0.89, 8.26]). There was no effect of credibility type on enrolling, χ^2^ (2) = 1.54, p = .46.

**Appendix 19:** Transparency structural

**Method:**

A sample of 512 researchers who were enrolled in the Rapid Response Network in April 2022 were randomized equally into two groups. The “transparency” group received email updates about what had happened with policy opportunities they had been emailed about previously. The control group did not receive such updates. We captured the number of times they had received an opportunity to engage as well as how many times they responded to the opportunity.

**Results:**

There was no effect of condition on the number of contributions (IRR = 0.68, SE = 0.18, z = -1.51, p = .13, 95%CI[0.41, 1.12]).

**Appendix 20:** Transparency MT

**Method:**

A sample of 524 researchers were randomized to receive either an email thanking them for their participation (control) or explaining some processes we frequently receive questions about (transparency). Data were captured and analyzed to test whether recipients opened or clicked the email.

| Control | Subject line: RPC: Thanks for your involvement  Dear {{appropriate_greeting}},  I hope this message finds you well. I wanted to send a brief thank you for your involvement in the Research-to-Policy Collaboration (RPC) Rapid Response Network. Your involvement helps us deliver on our commitment to strengthening the use of research in policy by facilitating meaningful, action-oriented collaboration between legislative officials and researchers. Our primary goal is to support the non-partisan use of scientific evidence by facilitating partnerships between researchers (including applied research/evaluation) and lawmakers.  We believe it is more fruitful to work within the context of existing policy priorities because our primary objective is to facilitate trusting partnerships between research and policy communities. Because the approach is non-partisan, we are less interested in how these problems are addressed so long as the methodologies are informed by research evidence and ultimately advance wellbeing. Therefore, we rarely take explicit stances on specific policies because, most often, there are multiple ways to approach a problem, and specific solutions tend to fall on party lines. We take an educational approach by synthesizing literature related to policies, without making a plea for one policy strategy over another.  You can read more about why this work is so important here: <https://research2policy.org/empirical-rationale/>  We appreciate your support of these efforts. Please reach out if I can answer any questions.  Sincerely, |
| --- | --- |
| Transparency | Subject line: RPC updates and looking to the future  Dear {{appropriate_greeting}},  I hope this message finds you well. I wanted to send a brief update about the current status of the Research-to-Policy Collaboration’s efforts, which aims to strengthen the use of research in policy by facilitating trusted relationships. Last summer, the RPC started recruiting researchers for the Rapid Response Network and over 500 researchers agreed! In the fall, we focused on creating a process to understand ways to improve our rapid response workflow. Then, over the winter, we hired policy associates to help implement the RPC’s non-partisan process, which is why you might have noticed an uptick in activity.  We anticipate that the most fruitful time for supporting congressional offices is now until elections heat up in August/September. Currently, we are focused on strengthening the rapid response process, but soon we will turn our attention to doing follow-up meetings with the 52 Congressional offices we’ve met with since last summer, and will ask some of the contributing researchers to join those meetings. We hope to provide more policy training materials later this year as well. Next year, with the new Congress, we will be ready to implement the full RPC model including more Hill Days that some researchers have really enjoyed in the past.  You can read about the Rapid Response process here: <https://research2policy.org/rapid-response-process/>  We appreciate your support of these efforts. Please reach out if I can answer any questions.  Sincerely, |

**Results:**

There was no effect of condition on whether someone opened at all (OR = 0.96, SE = 0.17, z = -0.26, p = .79, 95%CI[0.68, 1.35]) or how many times they opened (IRR = 1.24, SE = 0.19, z = 1.41, p = .16, 95%CI[0.92, 1.66]). There was also no effect of condition on whether someone clicked at all (OR = 1.29, SE = 0.38, z = 0.87, p = .38, 95%CI[0.73, 2.30]), or how many times they clicked (IRR = 1.53, SE = 0.52, z = 1.26, p = .21, 95%CI[0.79, 2.96]).
